# Viral *N*^6^-methyladenosine upregulates replication and pathogenesis of human respiratory syncytial virus

**DOI:** 10.1038/s41467-019-12504-y

**Published:** 2019-10-09

**Authors:** Miaoge Xue, Boxuan Simen Zhao, Zijie Zhang, Mijia Lu, Olivia Harder, Phylip Chen, Zhike Lu, Anzhong Li, Yuanmei Ma, Yunsheng Xu, Xueya Liang, Jiyong Zhou, Stefan Niewiesk, Mark E. Peeples, Chuan He, Jianrong Li

**Affiliations:** 1 Department of Veterinary Biosciences, College of Veterinary Medicine, The Ohio State University, Columbus, OH 43210 USA; 2 Department of Chemistry, Department of Biochemistry and Molecular Biology, and Institute for Biophysical Dynamics, The University of Chicago, Chicago, IL 60637 USA; 3 Center for Vaccines and Immunity, The Research Institute at Nationwide Children’s Hospital, Columbus, OH 43205 USA; 4 Institute of Translational Medicine, The First Affiliated Hospital of Wenzhou Medical University, Wenzhou, 325015 Zhejiang, P.R. China; 5 College of Animal Sciences, Zhejiang University, Hangzhou, 310058 Zhejiang, P. R. China; 6 Department of Pediatrics, The Ohio State University College of Medicine, Columbus, OH 43210 USA; 7 Howard Hughes Medical Institute, The University of Chicago, Chicago, IL 60637 USA

**Keywords:** RNA, Live attenuated vaccines, Virus-host interactions, Viral infection

## Abstract

*N*^6^-methyladenosine (m^6^A) is the most prevalent internal modification of mRNAs in most eukaryotes. Here we show that RNAs of human respiratory syncytial virus (RSV) are modified by m^6^A within discreet regions and that these modifications enhance viral replication and pathogenesis. Knockdown of m^6^A methyltransferases decreases RSV replication and gene expression whereas knockdown of m^6^A demethylases has the opposite effect. The G gene transcript contains the most m^6^A modifications. Recombinant RSV variants expressing G transcripts that lack particular clusters of m^6^A display reduced replication in A549 cells, primary well differentiated human airway epithelial cultures, and respiratory tracts of cotton rats. One of the m^6^A-deficient variants is highly attenuated yet retains high immunogenicity in cotton rats. Collectively, our results demonstrate that viral m^6^A methylation upregulates RSV replication and pathogenesis and identify viral m^6^A methylation as a target for rational design of live attenuated vaccine candidates for RSV and perhaps other pneumoviruses.

## Abstract (web summary)

Here, Xue et al. identify *N*^6^-methyladenosine (m^6^A) modification sites in RNAs of respiratory syncytial virus (RSV) and show that these sites, particularly sites in the transcript encoding for the viral glycoprotein, affect virus replication in primary human cells and cotton rats.

## Introduction

Internal *N*^6^-methyladenosine (m^6^A) is the most prevalent modification in the mRNA of most eukaryotes^1,2^. Although m^6^A was discovered in the 1970s^3–6^, its biological functions remained a mystery for decades. In 2011, m^6^A demethylases were discovered^7,8^. It is generally believed that m^6^A methylation is reversible although studies also suggest that a substantial degree of demethylation did not occur in some mRNA exons^9^. Transcriptome-wide mapping of m^6^A by high-throughput sequencing technology was reported subsequently^10–12^. A series of m^6^A-related enzymes and proteins were also discovered and characterized in mammalian cells. Recent work has found that m^6^A regulates RNA metabolism, protein translation, gene expression, and embryonic development in organisms ranging from plants to mice^13–17^.

The m^6^A modification is installed by the m^6^A methylation “writer” protein, a multicomponent S-adenosyl-l-methionine (SAM)-dependent methyltransferase complex, composed of two subunits, METTL3 (a catalytic enzyme) and METTL14 (an allosteric activator), and facilitated by the Wilms tumor 1 associated protein (WTAP)^18,19^. Interestingly, m^6^A modification can be selectively removed by two mammalian RNA demethylases, FTO (fat mass and obesity-associated protein) and its homolog, ALKBH5, both of which are termed m^6^A “eraser” proteins^7,8^. The biological functions of m^6^A are mediated through m^6^A “reader” proteins that specifically recognize and bind the methylated adenosine on RNA. The recently characterized m^6^A binding proteins include the YT521-B homology (YTH) domain family of proteins (YTHDF1, YTHDF2, YTHDF3, and YTHDC1)^16,20,21^. These m^6^A binding proteins regulate protein translation and RNA decay^22,23^.

As obligate intracellular parasites, viruses must synthesize their own genetic material and carry out their reproduction while avoiding innate immune surveillance by mimicking their host. Studies from the early 1970s showed that viral RNAs of several DNA viruses, retroviruses, and influenza virus contained internal m^6^A modifications^4,24–29^. Although it is still controversial whether viral m^6^A positively or negatively regulates HIV replication^30–32^, it was shown that m^6^A promotes gene expression of influenza virus^33^ and simian virus 40^34^. In the case of Kaposi’s sarcoma-associated herpesvirus (KSHV), the impact of m^6^A machinery on viral gene expression is dependent on the cell type^35–37^. In contrast, m^6^A negatively regulates the production of hepatitis C virus (HCV) and Zika virus^38^. However, the mechanism(s) by which m^6^A methylation regulates the virus life cycle remain poorly understood. In addition, the roles of m^6^A in viral virulence, pathogenesis, and immunity are not clear.

Non-segmented negative-sense (NNS) RNA viruses encompass a wide range of significant human, animal, and plant pathogens. For many of these agents, there are no effective vaccines or antiviral drugs. The replication and gene expression strategy of NNS RNA viruses is unique. During transcription, the polymerase transcribes the linear array of genes in the viral genome into 5–10 mRNAs that are capped and methylated at the 5′ end and polyadenylated at the 3′ end. During replication, the polymerase initiates at the extreme 3′ end of the genome and ignores the gene junctions to synthesize a full-length complementary antigenome which is encapsidated by N protein and subsequently serves as template for the synthesis of full-length progeny genomes that are likewise encapsidated by N protein^39,40^. A good example of NNS RNA viruses is human respiratory syncytial virus (RSV), a member of the *Pneumoviridae*^41^. RSV is the most important cause of upper and lower respiratory tract infection of infants, young children, and immunocompromised individuals and second only to influenza virus for the elderly^42^. Worldwide it is estimated that RSV causes 3.4 million hospitalizations and between 66,000 and 199,000 deaths in children <5 years of age^43^. Despite major efforts, no vaccine or antiviral drug is yet available for RSV^42^.

Here, we find that the RSV genome, antigenome, and mRNAs are m^6^A methylated internally at specific sites which positively regulate RSV replication, gene expression, and virus production in HeLa and A549 cells. Subsequently, the m^6^A sites in the viral *G* gene, the most abundant m^6^A enrichment gene, are mutated in an infectious cDNA clone of RSV. The resultant m^6^A deficient rgRSVs have significant defects in replication, gene expression, spread, and virus release in A549 cells and primary well differentiated human airway epithelial (HAE) cultures. These m^6^A mutated rgRSVs are defective in viral replication in the upper and lower respiratory tract of cotton rats and produce less pathology in the lungs. Importantly, we find that one m^6^A-mutated rgRSV is completely attenuated in cotton rats yet retains a wild-type level of immunogenicity. Collectively, these results reveal that m^6^A upregulates each step in the RSV replication cycle and viral pathogenesis, and identify m^6^A as a new target for the rational design of live attenuated vaccine candidates and antiviral drugs for RSV.

## Results

### The RSV genome and antigenome/mRNAs are m^6^A methylated

RSV has a NNS RNA genome of 15,222 nucleotides (RSV A2 strain). As is typical for NNS RNA viruses, replication of the viral genomic RNA (vgRNA) produces an exact, positive-sense full-length complementary RNA (cRNA) antigenome^44^. Both the genome and antigenome are encapsidated by the nucleocapsid (N) protein and both types of nucleocapsids can be packaged into virions, as for many NNS RNA viruses^45^. To investigate whether RSV RNA contains m^6^A, RNA was extracted from highly purified virions grown in HeLa cells, and the purity of RNA was examined by real-time RT-PCR to ensure that they were free from any contamination of host RNAs and viral mRNAs (Supplementary Fig. 1). The presence of m^6^A in viral RNA was quantified by liquid chromatography-tandem mass spectrometry (LC-MS/MS). We found that ~0.7% of the A bases were m^6^A methylated in RSV viral RNAs, a somewhat higher level than the host mRNAs (0.1–0.4%).

**Fig. 1:**
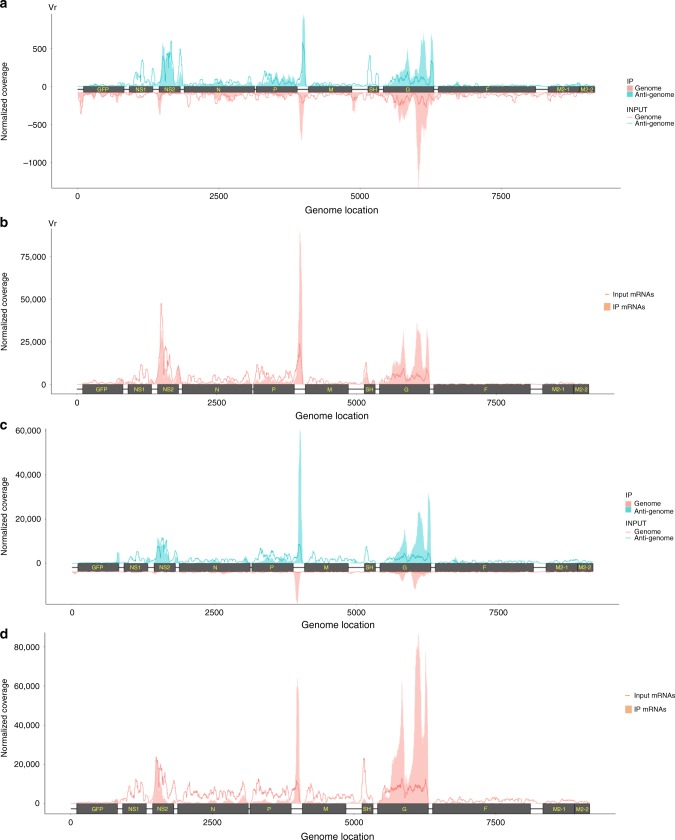
The RSV genome and antigenome/mRNAs are m^6^A methylated. **a** Distribution of m^6^A peaks in the RSV antigenome and genome of virions grown in HeLa cells. Confluent HeLa cells were infected by rgRSV at an MOI of 1.0, supernatant was harvested at 36 h post-infection. RSV virions were purified by sucrose gradient ultracentrifugation. Total RNAs were extracted from purified virions and were subjected to m^6^A-specific antibody immunoprecipitation followed by high-throughput sequencing (m^6^A-seq). A schematic diagram of partial RSV antigenome (complementary to regions from the leader sequence to *M2-2* gene) is shown, as most m^6^A peaks are clustered in these regions. m^6^A sites in full-length antigenome and genome are shown in Supplementary Fig. 2. The normalized coverage from m^6^A-seq of RSV RNA showing the distribution of m^6^A-immunoprecipitated (IP) reads mapped to the RSV antigenome (blue block) and genome (pink block). The baseline distributions for antigenome and genome from input sample are shown as a blue and pink line respectively. Data presented are the averages from two independent virion samples (*n* *=* 2). **b** Distribution of m^6^A peaks in the RSV mRNAs from RSV-infected HeLa cells. Confluent HeLa cells were infected by rgRSV at an MOI of 1.0, cell lysates were harvested at 36 h post-infection. Total RNAs were extracted from cell lysates, and were enriched for mRNA by binding to oligo dT, and subjected to m^6^A-seq. The distribution of m^6^A-immunoprecipitated (IP) reads were mapped to the RSV mRNAs (pink block). The baseline distributions for mRNAs from input sample are shown as a pink line. Data presented are the averages from two independent virus-infected HeLa cell samples (*n* *=* 2). **c** Distribution of m^6^A peaks in the RSV antigenome and genome of virions grown in A549 cells. Data presented are the averages from two independent virion samples (*n* *=* 2). **d** Distribution of m^6^A peaks in the RSV mRNAs from RSV-infected A549 cells. Data presented are the averages from two independent virus-infected A549 cell samples (*n* *=* 2)

To locate the m^6^A sites on RSV RNA, we sonicated virion RNA and subjected it to m^6^A-specific antibody immunoprecipitation followed by high-throughput sequencing (m^6^A-seq), then mapped all the reads onto either the genome or antigenome sequence. Several m^6^A peaks were identified on both strands of the viral RNA (Supplementary Fig. 2A and Fig. 1a). The RSV antigenomic RNA contained major m^6^A peaks in the regions complementary to the *N*, *P*, *G*, and *F* genes and in the regions complementary to the two regulatory elements, the gene end (*ge*) sequence of *N* and the intergenic (*ig*) sequence between the *P* and *M* genes in the genome (Fig. 1a, and Supplementary Fig. 2A and Supplementary Table 1). In the genomic RNA, eleven m^6^A peaks were detected in the *NS2*, *N*, *P*, *M*, *G*, and *L* genes and four regulatory elements including the gene start (*gs*) of *NS2*, *ig* between *P* and *M*, *ge* of *M*, *ig* between *M* and *SH*, *ge* of *G*, and *ig* between *G* and *F* (Fig. 1a and Supplementary Fig. 2A and Supplementary Table 1). Since we used a recombinant RSV harboring GFP between the leader and the *NS1* gene (rgRSV), we also searched whether GFP region contains m^6^A. An m^6^A peak with a size of 60 nt was detected in *GFP* gene in genome (Supplementary Table 1). No m^6^A peak was found in GFP region in antigenome. The *G* gene regions from both genome and antigenome have the strongest m^6^A enrichment with peak size of 822 nt and 672 nt, respectively, indicating that there may be multiple adjacent m^6^A sites in these regions. Together, these results confirm that both RSV genome and antigenome RNAs contain m^6^A.

We also mapped m^6^A peaks in mRNAs purified from RSV-infected cells. To do this, total RNA was isolated from rgRSV-infected HeLa cells, enriched for mRNA by binding to oligo dT, and subjected to m^6^A-seq. As RSV mRNAs contain poly(A) and are subsequently detected from poly(dT)-enriched m^6^A-seq, we identified 16 m^6^A peaks from RSV mRNAs (Fig. 1b, Supplementary Fig. 2B and Supplementary Table 2) which largely overlapped with those of the antigenome (Fig. 1a, Supplementary Fig. 2A and Supplementary Table 1). Interestingly, the *G* gene transcript has the strongest m^6^A enrichment with a 846 bp peak size. In addition, no m^6^A peak was detected in GFP mRNA in virus-infected HeLa cells.

We next performed m^6^A-seq of rgRSV grown in A549 cells, a relevant cell line for RSV infection. Similar to HeLa cells, we found that RSV genome, antigenome, and mRNAs were m^6^A methylated in A549 cells **(**Fig. 1c, d, and Supplementary Fig. 2C, D**)**. For virion RNAs, a total of 9 and 15 m^6^A peaks were identified in the genome and antigenome, respectively (Fig. 1c and Supplementary Fig. 2C and Supplementary Table 3). Similar to virions grown in HeLa cells, the location of m^6^A peaks identified from genome and antigenome largely overlap. *G* gene regions from both genome and antigenome have the strongest m^6^A enrichment with 696 and 846 bp peak size, respectively. An m^6^A peak was detected in *GFP* gene in antigenome but no m^6^A peak was detected in *GFP* gene in genome. For the RNAs extracted from virus-infected cells, a total of 18 m^6^A peaks were identified in RSV mRNAs (Fig. 1d and Supplementary Fig. 2D and Supplementary Table 4). Again, the *G* gene transcript has the strongest m^6^A enrichment with 1046 bp peak size. In addition, one m^6^A peak was found in GFP mRNA in infected A549 cells.

We next analyzed the overlapping regions based on m^6^A-seq data from HeLa and A549 cells (Supplementary Tables 5 and 6). For virion RNA, six and four overlapping regions were identified in the genome (*gs* of *NS2*, *NS2*, *N*, *P*, *ig* between *P* and *M*, and *G*) and antigenome (*N*, *M*, *G*, and *F*), respectively (Supplementary Table 5). For RNAs purified from RSV-infected cells, 11 overlapping m^6^A peaks were also found in mRNAs, respectively (Supplementary Table 6). Although there are some differences, the majority of m^6^A peaks are highly conserved between the two cell lines suggesting that RSV utilizes the host m^6^A machinery to methylate these specific sites.

### RSV infection alters the m^6^A distribution of host RNAs

We next determined the effects of RSV infection on the abundance and distribution of m^6^A on cellular transcripts. Metagene analysis showed that RSV-infected and mock-infected HeLa cells have m^6^A peaks enriched near the start and stop codons of open reading frames (Supplementary Fig. 3A), which is consistent with the known distribution of m^6^A sites on transcripts^10–12^. Unlike the distribution of m^6^A peaks on mRNA, the peaks are mostly uniformly distributed on lncRNA with a slightly more enrichment at its 5′ end (Supplementary Fig. 3B). The distribution of m^6^A peaks in each annotation also recapitulate m^6^A site distribution^10–12^ with the majority of peaks residing in the CDS and 3′ UTR regions (Supplementary Fig. 3C, D). Differential peak analysis using the count based QNB test^46^ identified 2256 differentially methylated peaks (Supplementary Data 1). Analysis of RNA-seq data from the host cell (HeLa) revealed over 9000 differentially expressed genes at an adjusted *P* value cutoff of 0.05 (Supplementary Data 2). These data suggest RSV infection significantly altered both the epitranscriptome and the transcriptome of the host cells. Pathway enrichment analysis shows differentially expressed genes are enriched in pathways including cell cycle, metabolism, autophagy, RNA synthesis and transport, and response to viral infection (Supplementary Fig. 3E).

As expected, the distribution of m^6^A sites was highly conserved between cell lines (Supplementary Fig. 4A–D). RSV infection altered the expression of over 7000 host cell genes in A549 cells (Supplementary Data 3) involved in a series of signal pathways (Supplementary Fig. 4E) but very few m^6^A peaks were found to be differentially methylated (Supplementary Data 4). We also analyzed the differentially expressed genes which are overlapped in pathways between the HeLa and A549 cells. Many of these overlapped differentially expressed genes are involved cell cycle, metabolism, TNFα signaling, and RNA transport (Supplementary Fig. 4F**)**. Therefore, RSV infection may have widespread effects on host gene expression partially attributed to alteration of the deposition of m^6^A.

### m^6^A reader proteins positively regulate RSV replication

**Fig. 2:**
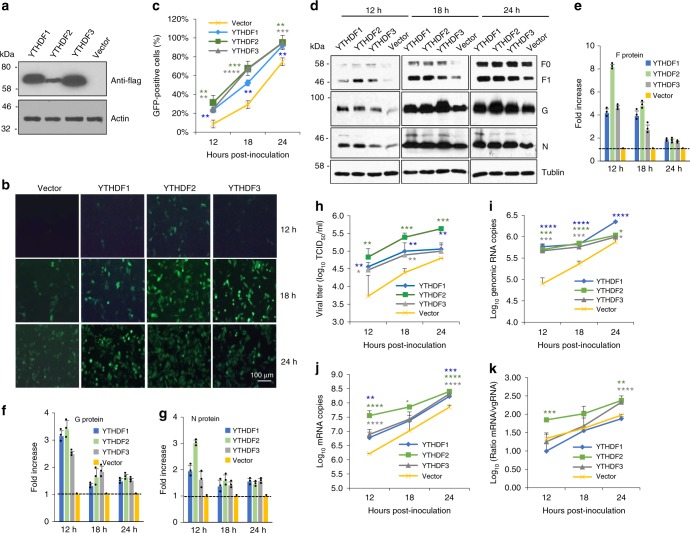
YTHDF1, 2, 3 (reader) proteins promote RSV replication, gene expression, and progeny virus production. **a** Detection of YTHDF1, 2, 3 in HeLa cells stably overexpressing YTHDF1-3. Western blot confirmed the overexpression of YTHDF1-3 proteins in HeLa cells using anti-Flag antibody. **b** YTHDF1, 2, 3 enhance GFP expression in rgRSV-infected cells. HeLa cells stably overexpressing these YTHDF proteins were infected with rgRSV at an MOI of 0.1, and GFP expression was monitored at the indicated times by fluorescence microscopy. Micrographs with ×10 magnification (scale bar of 100 μm) are shown. **c** YTHDF1, 2, 3 increase the number of GFP-positive cells quantified by flow cytometry. The *P* value (Student’s *t*-test) for YTHDF1 at 12, 18, and 24 h is **P = 0.00297, **P = 0.00145, and **P = 0.00202, respectively; for YTHDF2 at 12, 18, and 24 h is **P = 0.00318, ***P = 0.000892, and **P = 0.00312, respectively; for YTHDF3 at 12, 18, and 24 h is **P = 0.00119, ****P = 5.19 × 10^−5^, and ***P = 0.000647, respectively. **d** YTHDF1, 2, 3 enhance RSV protein expression. Total cell extracts were harvested from rgRSV-infected HeLa cells at the indicated times and subjected to western blot using antibody against RSV N, F, or G protein. Western blots shown are the representatives of three independent experiments. RSV F (F0 + F1) (**e**), G (**f**), and N (**g**) proteins were quantified by Image J Software. Data are expressed as mean of three independent experiments ± standard deviation. **h** YTHDF1, 2, 3 increase RSV progeny virus production. The release of infectious RSV particles was monitored by a single-step growth curve. Virus titer was measured by TCID_50_. The *P* value (Student’s *t*-test) for YTHDF1 at 12, 18, and 24 h is **P = 0.0349, **P = 0.00797, and **P = 0.00741, respectively; for YTHDF2 at 12, 18, and 24 h is **P = 0.0176, ***P = 0.000279, and ***P = 1.58 × 10^−25^, respectively; for YTHDF3 at 12 and 18 h is *P = 0.0487 and **P = 0.00317, respectively. **i** YTHDF1, 2, 3 enhance RSV genomic RNA replication. Total RNA was purified from rgRSV-infected cells using TRizol, and genomic RNA was quantified by real-time RT-PCR using specific primers annealing to the RSV leader sequence and *GFP* gene. The *P* value (Student’s *t*-test) for YTHDF1 at 12, 18, and 24 h is ****P = 3.68 × 10^−5^, ****P = 5.46 × 10^−5^, and ****P = 4.96 × 10^−5^, respectively; for YTHDF2 at 12, 18, and 24 h is ***P = 0.000237, ****P = 1.48 × 10^−5^, and *P = 0.0184, respectively; for YTHDF3 at 12, 18, and 24 h is ***P = 0.000165, ***P = 0.000128, and *P = 0.0475, respectively. **j** YTHDF1, 2, 3 enhance mRNA transcription. Viral mRNA was separated from total RNA using the Dynabeads mRNA isolation kit and quantified by real-time PCR using primers annealing to the *NS1* gene. The *P* value (Student’s *t*-test) for YTHDF1 at 12 and 24 h is **P = 0.00794 and ***P = 0.000218, respectively; for YTHDF2 at 12, 18, and 24 h is ****P = 4.94 × 10^−6^, *P = 0.0430, and ****P *=* 3.46 × 10^−5^, respectively; for YTHDF3 at 12 and 24 h is ****P = 5.93 × 10^−6^ and ****P *=* 2.39 × 10^−5^, respectively. **k** Ratio between mRNA and genomic RNA. The ratio between NS1 mRNA and genomic RNA was calculated for each cell line. All results are from three independent experiments. Flow cytometry data are expressed as mean of three independent experiments ± standard deviation. RNA copy and viral titer are the geometric mean titer (GMT) of three independent experiments ± standard deviation. The *P* value (Student’s *t*-test) for YTHDF2 at 12 and 24 h is ***P = 0.000601 and **P = 0.00130, respectively; for YTHDF3 at 24 h is ****P = 0.000599. Data were analyzed using Student’s *t*-test and statistical differences were indicated as *P < 0.05; **P < 0.01; ***P < 0.001; and ****P < 0.0001

To begin to explore the role of m^6^A modification in RSV replication and gene expression, we first took advantage of HeLa cells that stably overexpress m^6^A “reader” proteins, YTHDF1, YTHDF2, and YTHDF3 (Fig. 2a). As shown in Fig. 2b, stronger GFP expression (more green cells, brighter cells) was observed in HeLa cells overexpressing YTHDF1-3 compared to the vector control. Quantification by flow cytometry showed that significantly more GFP-positive cells and higher GFP density were detected in HeLa cells overexpressing m^6^A reader proteins than in the vector control (*P* < 0.05) (Fig. 2c and Supplementary Fig. 5).

We next measured the expression of RSV F, G, and N proteins. As shown in Fig. 2d, more F, G, and N proteins were detected in all three YTHDF-overexpressing HeLa cell lines. Quantitative analysis showed a dramatic increase in viral protein expression during the first 12 h although later time points were not as large (Fig. 2e–g). Next, we measured the release of infectious virus particles in a single-step growth curve. The RSV titer was significantly increased in all three YTHDF-overexpressing cell lines (Fig. 2h) (*P* < 0.05 or 0.01). Overexpression of YTHDF2 had the most dramatic impact on virus production, increasing RSV titer by 1–2 logs compared to the vector control HeLa cells (*P* < 0.05 or 0.01) (Fig. 2h).

The upregulating role of m^6^A reader proteins on RSV replication was also confirmed in HeLa cells transfected with plasmids expressing YTHDF1-3 (Supplementary Fig. 6A). Compared to HeLa cells stably overexpressing YTHDF1-3 (Fig. 2d), transient overexpression of YTHDF1-3 led to a more robust enhancement of F and G protein synthesis (Supplementary Fig. 6B) and GFP expression (Supplementary Fig. 6C).

We further analyzed viral replication and gene expression in A549 cells. Similar to the observations in HeLa cells, enhanced F, G, and N protein synthesis (Supplementary Fig. 7A) and GFP expression (Supplementary Fig. 7B) was detected when YTHDF1-3 proteins were overexpressed. We also tested RSV replication in Vero cells, the WHO-approved cell line for production of RSV live attenuated vaccine candidates. Similarly, m^6^A reader protein (YTHDF1) enhanced RSV protein synthesis in Vero cells (Supplementary Fig. 7C**)**. Thus, we observed a pro-viral function for m^6^A in all three cell lines. It should be noted that overexpression of YTHDF1 protein (a representative of reader proteins) in A549 cells did not significantly affect the growth or survival of the cells (Supplementary Fig. 8A, B).

We also measured the RSV genomic RNA (the replication product) and mRNAs (the transcription product) in HeLa cells by real-time RT-PCR. Overexpression of YTHDF1-3 significantly increased both RSV genomic RNA (Fig. 2i) and mRNA synthesis (Fig. 2j). Overexpression of YTHDF1 and 3 did not alter the balance between the synthesis of genomic RNA and mRNA whereas overexpression of YTHDF2 led to a more dramatic increase in replication than transcription (Fig. 2k). It appears that overexpression of YTHDF1-3 enhanced the ability of the RSV polymerase to both replicate and transcribe.

**Fig. 3:**
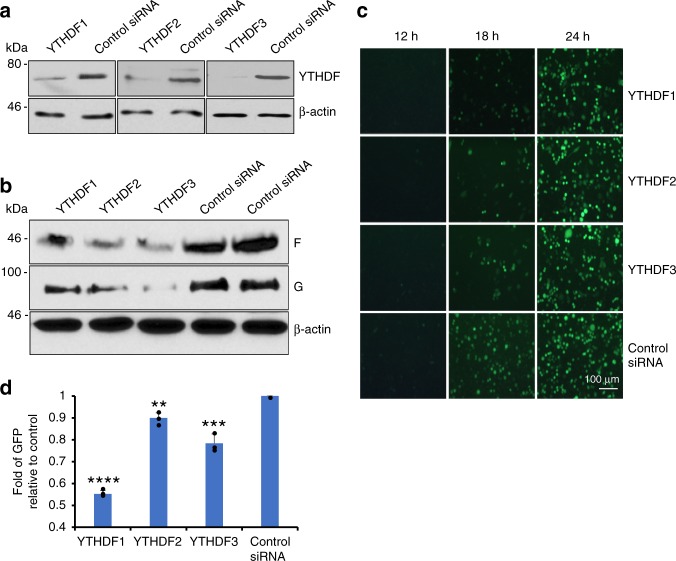
Knockdown of endogenous YTHDF1, 2, 3 (reader) proteins diminishes RSV gene expression. HeLa cells were transfected with 150 pmole of siRNA targeting YTHDF1, 2, 3 or control siRNA. At 36 h post-transfection, cells were infected with rgRSV at an MOI of 0.5. **a** Immunoblot analysis of YTHDF1, 2, 3 in HeLa cells transfected with siRNA. **b** Immunoblot analysis of RSV G and F proteins. **c** Dynamics of GFP expression in YTHDF1, 2, 3 protein-depleted HeLa cells. Micrographs with ×10 magnification (scale bar of 100 μm) are shown. **d** Quantification of GFP-positive cells by flow cytometry at 18 h post-inoculation. Fold of GFP signal compared to the control is shown. Western blots and GFP images shown are the representatives of three independent experiments. Flow cytometry data are expressed as mean of three independent experiments ± standard deviation. The *P* value (Student’s *t*-test) for YTHDF1, 2, and 3 is ****P = 1.10 × 10^−6^, **P = 0.00170, and ***P = 0.000972, respectively

As a complementary approach, we also tested RSV replication and gene expression in HeLa cells when m^6^A reader proteins were knocked down by siRNA. Counting live cells by flow cytometry showed that siRNA targeting YTHDF1 did not significantly alter cell survival (Supplementary Fig. 8C, D). Knockdown of individual, endogenous YTHDF1-3 proteins (Fig. 3a) significantly reduced viral F and G protein synthesis (Fig. 3b) and GFP expression (Fig. 3c, d) relative to the control siRNA transfected cells. Collectively, these results demonstrate that m^6^A binding proteins promote RSV genome replication, mRNA transcription, and as a result, viral protein expression, and progeny virus production.

### m^6^A writer proteins positively regulate RSV replication

**Fig. 4:**
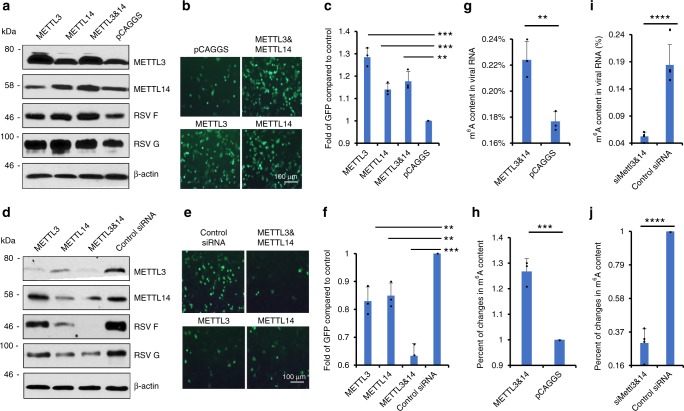
Effects of m^6^A writer proteins on RSV gene expression. **a** Overexpression of m^6^A writer proteins enhances RSV gene expression. HeLa cells were transfected with plasmids encoding METTLE3 and/or METTL14, followed by rgRSV infection at an MOI of 0.5. At 18 h post-infection, cell lysates were harvested for western blot analysis. **b** Overexpression of m^6^A writer proteins enhances RSV expression of GFP. The GFP expression was monitored by fluorescence microscopy. Representative micrographs with ×10 magnification (scale bar of 100 μm) at 18 h post-infection were shown. **c** Quantification of GFP-positive cells by flow cytometry at 18 h post-infection. Fold of GFP signal compared to the control is shown. The *P* value (Student’s *t*-test) for METTL3, METTL14, and METTL3 & METTL14 is ***P = 0.000276, ***P = 0.000873, and **P = 0.00228, respectively. **d** Knockdown of m^6^A writer proteins diminishes RSV gene expression. HeLa cells were transfected with siRNA targeting METTL3 and/or METTL14, followed by rgRSV infection at an MOI of 0.5. At 18 h post-infection, cell lysates were harvested for western blot analysis. **e** Knockdown of m^6^A writer proteins diminishes GFP expression. Micrographs with ×10 magnification (scale bar of 100 μm) are shown. **f** Quantification of GFP-positive cells by flow cytometry. Fold of GFP signal compared to the control is shown. Flow cytometry data are expressed as mean ± standard deviation. The *P* value (Student’s *t*-test) for METTL3, METTL14, and METTL3 & METTL14 is **P = 0.00441, **P = 0.00458, and ****P = 0.000134, respectively. **g** Overexpression of m^6^A writer proteins enhances m^6^A level in viral RNA. Confluent A549 cells were transfected with plasmids encoding Mettl3 and Mettl14 or vector pCAGGS, followed by rgRSV infection. RSV particles were harvested and purified. The m^6^A content in viral RNA was determined as described in Materials and Methods. The *P* value (Student’s *t*-test) is **P = 0.00684. **h** Fold increase in m^6^A content in viral RNA compared to controls. The *P* value (Student’s *t*-test) is ***P = 0.000764. **i** Knockdown of m^6^A writer proteins reduces m^6^A level in viral RNA. A549 cells were transfected with siRNA targeting METTL3 and METTL14 or control siRNA, followed by rgRSV infection. RSV particles were harvested and purified. The m^6^A content in viral RNA was determined. The *P* value (Student’s *t*-test) is ****P = 1.29 × 10^−7^. **j** Fold reduction in m^6^A content in viral RNA compared to controls. The *P* value (Student’s *t*-test) is ****P = 2.45 × 10^−12^. Results are from three or four independent experiments

The internal m^6^A addition is catalyzed by host methyltransferases termed m^6^A writer proteins (METTL3 and METTL14)^18^. We next examined the role of the m^6^A writer proteins in RSV replication and protein expression. More F and G protein synthesis (Fig. 4a) and GFP expression (Fig. 4b, c) were observed when METTL3 and METTL14 were overexpressed in HeLa cells. In contrast, less F and G proteins were synthesized (Fig. 4d) and less GFP was expressed (Fig. 4e, f) when endogenous METTL3, METTL14, or both, were knocked down. siRNA targeting METTL3 did not significantly alter cell survival (Supplementary Fig. 8C, D). These results suggest that modification of RSV RNA by m^6^A writers facilitates RSV replication and gene expression.

Next, we determined whether alteration of m^6^A writer proteins can alter the level of m^6^A level in viral RNA. As shown in Fig. 4g, h, the m^6^A contents in viral RNA of virus from METTL3 and METTL14-overexpressed cells were significantly higher (~26% increase) than viral RNA of particles from vector-transfected cells. Knockdown of METTL3 and  METTL14 led to 70% of reduction in m^6^A content in viral RNA (Fig.4i, j). These experiments suggest that manipulation of m^6^A writer proteins lead to enhanced or reduced levels of m^6^A in viral RNAs, which may in turn, affect viral replication and gene expression.

### m^6^A eraser proteins downregulated RSV replication

**Fig. 5:**
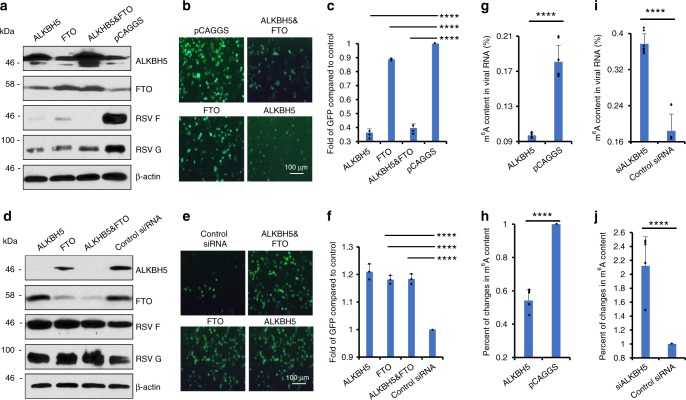
Effects of m^6^A eraser proteins on RSV gene expression. **a** Overexpression of m^6^A eraser proteins diminishes RSV gene expression. HeLa cells were transfected with plasmids encoding ALKBH5 and/or FTO, followed by rgRSV infection at an MOI of 0.5. At 18 h post-infection, cell lysates were harvested for Western blot analysis. **b** Overexpression of m^6^A eraser proteins reduces GFP expression. The GFP expression was monitored by fluorescence microscopy. Representative micrographs with 10 × magnification (scale bar of 100 μm) at 18 h post-infection were shown. **c** Quantification of GFP-positive cells by flow cytometry at 18 h post-infection. The *P* value (Student’s t-test) for ALKBH5, FTO, and ALKBH5&FTO is ****P = 1.91 × 10^−6^, ****P = 1.34 × 10^−5^, and ****P = 3.61 × 10^−6^, respectively. **d** Knockdown of m^6^A eraser proteins enhances RSV gene expression. HeLa cells were transfected with siRNA targeting ALKBH5 and/or FTO, followed by rgRSV infection at an MOI of 0.5. At 18 h post-infection, cell lysates were harvested for Western blot analysis. **e** Knockdown of m^6^A eraser proteins enhances GFP expression. Micrographs with 10 × magnification (scale bar of 100 μm) are shown. **f** Quantification of GFP-positive cells by flow cytometry. Fold of GFP signal compared to the control is shown. Western blots and GFP images shown are representatives of three independent experiments. Flow cytometry data are expressed as mean ± standard deviation. The *P* value (Student’s *t*-test) for ALKBH5, FTO, and ALKBH5&FTO is ****P = 2.06 × 10^−4^, ****P = 3.38 × 10^−5^, and ****P = 6.50 × 10^−6^, respectively. **g** Overexpression of m^6^A eraser protein reduces m^6^A level in viral RNA. A549 cells were transfected with plasmid encoding ALKBH5 or pCAGGS, followed by rgRSV infection. RSV particles were purified from supernatants. Virion RNA was subjected to m^6^A-IP. The *P* value (Student’s *t*-test) is ****P = 5.81 × 10^−9^. **h** Fold reduction in m^6^A content in viral RNA compared to controls. The *P* value (Student’s *t*-test) is ****P = 1.17 × 10^−11^. **i** Knockdown of m^6^A eraser protein increases m^6^A level in viral RNA. A549 cells were transfected with siRNA targeting ALKBH5 or control siRNA, followed by rgRSV infection. RSV particles were purified. Virion RNA was subjected to m^6^A-IP. The *P* value (Student’s *t*-test) is ****P = 5.09 × 10^−9^. **j** Fold increase in m^6^A content in viral RNA compared to controls. The *P* value (Student’s *t*-test) is ****P = 2.57 × 10^−6^. Results are from three or four independent experiments

Internal m^6^A modifications are reversible and can be removed by m^6^A eraser proteins^7,8^. We thus examined the effects of overexpression of eraser proteins ALKBH5 or FTO, or both (Fig. 5). Overexpression of eraser proteins dramatically reduced RSV F and G protein expression by 80- and 20-fold, respectively (Fig. 5a), and number of GFP cells by 1.3–2.7 times (Fig. 5b, c). Next, we knocked down ALKBH5 or FTO, or both, in HeLa cells, followed by rgRSV infection. siRNA targeting ALKBH5 did not significantly alter cell survival (Supplementary Fig. 8C, D). Knockdown of ALKBH5 and FTO enhanced the expression of F protein by 3-fold, G protein by 5-fold (Fig. 5d), and number of GFP cells by 1.2-fold (Fig. 5e, f) compared to the cells transfected with control siRNA. Therefore, overexpression of m^6^A eraser proteins negatively regulated RSV replication and gene expression. We also determined whether overexpression and knockdown of eraser proteins can affect m^6^A level of viral RNA. As shown in Fig. 5g, h, the m^6^A contents in viral RNA of virus from ALKBH5-overexpressed cells led to 46% reduction. Knockdown of ALKBH5 led to 2.12-fold increases in m^6^A abundance in viral RNA (Fig. 5i, j**)**. Therefore, manipulation of m^6^A eraser proteins can directly affect m^6^A levels in viral RNAs, which may affect viral replication and gene expression.

### RSV infection does not alter the translocation of m^6^A proteins

**Fig. 6:**
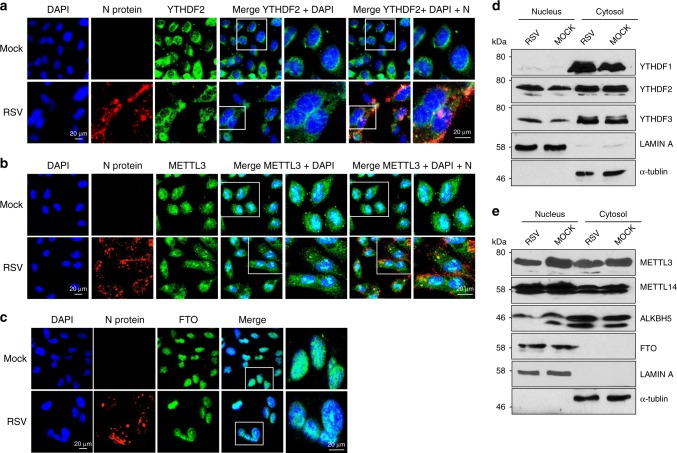
RSV infection does not alter the m^6^A reader, writer, or eraser protein distribution in cells. HeLa cells were infected by rgRSV at an MOI of 10.0. At 24 h post-infection, mock- or rgRSV-infected cells were stained with anti-reader, writer, or eraser protein antibody (green) and anti-RSV N protein antibody (red), and were analyzed by confocal microscope. Nuclei were labeled with DAPI (blue). Micrographs with ×60 magnification (scale bar of 20 μm) are shown. **a** m^6^A reader protein YTHDF2; **b** m^6^A writer protein METTL3; and **c** m^6^A eraser protein FTO. **d** Detection of m^6^A reader, writer, and eraser proteins by Western blot. Nuclear and cytoplasmic fractions were separated from mock- or rgRSV-infected HeLa cells, and were subjected to western blot. Nuclear and cytoplasmic markers were indicated by Lamin A and α-Tubulin, respectively. Representative results from three independent experiments are shown

To directly visualize the locations of the m^6^A reader, writer, and eraser proteins, mock and rgRSV-infected HeLa cells were stained with antibodies specific to each m^6^A-related protein and analyzed by confocal microscopy. As shown in Fig. 6a and Supplementary Fig. 9, m^6^A reader proteins (YTHDF1-3) were distributed in the cytoplasm in both mock and RSV-infected cells. In addition, viral N protein was partially co-localized with reader protein (YTHDF2) (Fig. 6a). In contrast, the majority of m^6^A writer proteins (METTL3 and METTL14) and eraser protein (ALKBH5) were distributed in the nucleus although a small fraction of these proteins was also found in the cytoplasm (Fig. 6b and Supplementary Figs. 10 and 11). Also, viral N protein was partially co-localized with writer protein (METTL3) (Fig. 6b). Another eraser protein, FTO, was exclusively located in the nucleus (Fig. 6c). Equal amounts of m^6^A related proteins were detected in the cytoplasmic and nuclear fractions by Western blot (Fig. 6d, e). Therefore, RSV infection does not significantly alter the distribution pattern of m^6^A-related proteins in HeLa cells.

### m^6^A reader proteins bind to both RSV genomic RNA and mRNA

Since the biological function of m^6^A is mediated by m^6^A binding proteins, we next determined whether YTHDF2 can directly bind to RSV RNAs in virus-infected cells. As expected, YTHDF2 was pulled down by YTHDF2-specific antibody (Supplementary Fig. 12A). Both RSV genomic RNA and N mRNA were efficiently precipitated as complexes, with YTHDF2 (Supplementary Fig. 12B). We further confirmed this result by pulling down HA-tagged YTHDF2 from total cell lysates of HeLa cells overexpressing YTHDF2 with HA antibody (Supplementary Fig. 12C). Similarly, significant amounts of RSV genomic RNA and N mRNA bound to YTHDF2 (Supplementary Fig. 12D). Since G mRNA has the strongest m^6^A enrichment, we also determined the binding of m^6^A reader proteins to G mRNA. All reader proteins (YTHDF1, 2, and 3) were capable of binding to G mRNA (Supplementary Fig. 12E).

### Abrogation of m^6^A sites results in attenuation of RSV

**Fig. 7:**
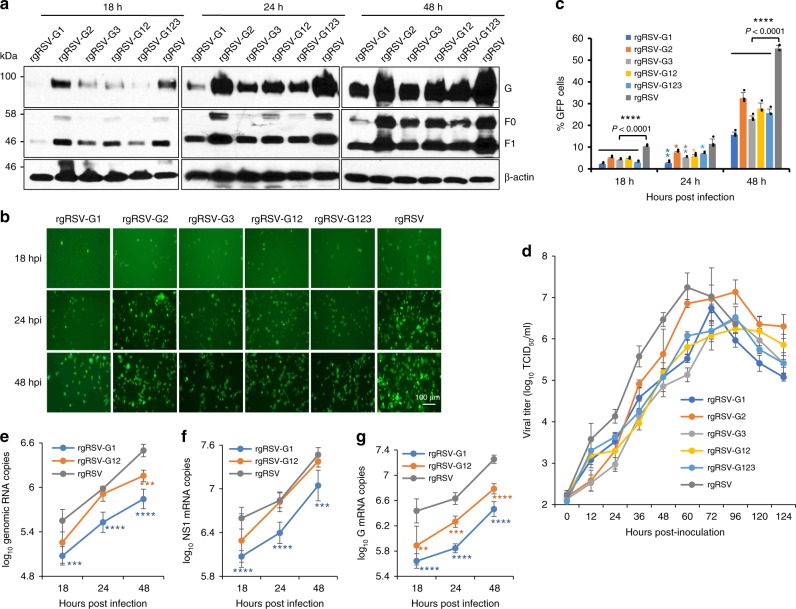
m^6^A-abrogating RSV mutants have defects in replication in immortalized cells. **a** Immunoblot analysis of RSV proteins. Confluent A549 cells were infected with each rgRSV at an MOI of 0.1, cell lysates were harvested at 18, 24, and 48 h post-infection, and RSV proteins were detected by specific antibodies against F and G protein. **b** GFP expression of m^6^A-deficient rgRSV mutants. Micrographs with ×10 magnification (scale bar of 100 μm) are shown. **c** Quantification of GFP-positive cells by flow cytometry. The *P* value (Student’s *t*-test) for rgRSV-G1, G2, G3, G12, and G123 at 18 h post-inoculation is ****P = 5.87 × 10^−6^, ****P = 7.13 × 10^−5^, ****P = 1.65 × 10^−5^, ****P = 6.49 × 10^−5^, and ****P = 6.98 × 10^−6^, respectively; at 24 h post-inoculation is **P = 0.00261, *P = 0.0418, **P = 0.00766, *P = 0.0138, and *P = 0.0230 respectively; at 48 h post-inoculation is ****P = 3.55 × 10^−6^, ****P = 1.82 × 10^−4^, ****P = 9.83 × 10^–6^, ****P = 7.78 × 10^−5^, and ****P = 2.48 × 10^−5^, respectively. **d** Single-step growth curve of m^6^A-deficient rgRSV mutants in A549 cells. Confluent A549 cells were infected with each rgRSV at an MOI of 1.0, supernatants were harvested, and viral titer was determined by TCID_50_ assay. **e** RSV genomic RNA replication. At 18, 24, and 48 h post-infection, total RNA was purified from rgRSV-infected cells using TRizol, and genomic RNA was quantified by real-time RT-PCR using specific primers annealing to the RSV leader sequence and GFP gene. The *P* value (Student’s *t*-test) for rgRSV-G1 at 18, 24, and 48 h is ***P = 0.000653, ****P = 8.66 × 10^−5^, and ****P = 1.33 × 10^−5^ respectively. The *P* value for rgRSV-G12 at 48 h is ***P = 0.000141. **f** RSV NS1 mRNA transcription. Viral mRNA was separated from total RNA and quantified by real-time PCR using primers annealing to the NS1. The *P* value (Student’s *t*-test) for rgRSV-G1 at 18, 24, and 48 h is ****P = 1.80 × 10^−5^, ****P = 1.11 × 10^−4^, and ***P = 0.001, respectively. **g** RSV G mRNA transcription. The *P* value (Student’s *t*-test) for rgRSV-G1 at 18, 24, and 48 h is ****P = 7.13 × 10^−5^, ****P = 5.10 × 10^−6^, and ****P = 1.22 × 10^−6^, respectively. The *P* value (Student’s *t*-test) for rgRSV-G12 at 18, 24, and 48 h is **P = 0.00942, ***P = 0.000199, and ****P = 1.35 × 10^−5^, respectively. Results are from three independent experiments. Flow cytometry data are expressed as mean ± standard deviation. RNA copy and viral titer are the geometric mean titer (GMT) of three independent experiments ± standard deviation. Western blots shown are the representatives of three independent experiments

Based on m^6^A-seq, G gene and G mRNA have the most abundant m^6^A enrichment in both HeLa and A549 cells (Fig. 1). Thus, we decided to mutate the m^6^A sites in the *G* gene region which we found to be conserved in the m^6^A-seq from both HeLa and A549 cells (Supplementary Fig. 13). These mutations were predicted to remove the m^6^A sites in the G mRNA and in the *G* gene region of the antigenome without changing the amino acid they encoded. Recombinant RSVs carrying these mutations were recovered by reverse genetics system. First, the potential m^6^A sites in regions 1, 2, and 3 were mutated individually to produce rgRSV-G1, G2, and G3, respectively. Second, mutations of m^6^A sites in regions 1 and 2 were combined to produce rgRSV-G12. Third, m^6^A sites in regions 1, 2, and 3 were combined to produce rgRSV-G123. All m^6^A-mutated rgRSVs had various degrees of reduction in viral F and G protein synthesis in A549 cells compared to the parental rgRSV (Fig. 7a). Significantly less GFP expression was observed in m^6^A-mutated rgRSVs compared to rgRSV at 48 h post-infection (Fig. 7b, c, and Supplementary Fig. 14). Single-step growth curves showed that m^6^A-mutated rgRSVs had delayed replication kinetics and had 0.5–1.5 log reductions in peak titer compared to rgRSV (Fig. 7d). Overall, mutants rgRSV-G1, G3, G12, and G123 had a moderate defect whereas rgRSV-G2 had a mild defect in replication. Real-time RT-PCR results showed that both rgRSV-G1 and G12 had defects in genome (Fig. 7e), NS1 (Fig. 7f), and G (Fig. 7g) mRNA synthesis compared to rgRSV, and rgRSV-G1 had more defects than rgRSV-G12. Next, we calculated the percentage of reduction for NS1 and G mRNA. It was found that NS1 mRNA had significantly less reduction than G mRNA, suggesting that removal of the m^6^A from the G mRNA may accelerate its decay.

### m^6^A-mutated rgRSVs are defective in replication in HAE

**Fig. 8:**
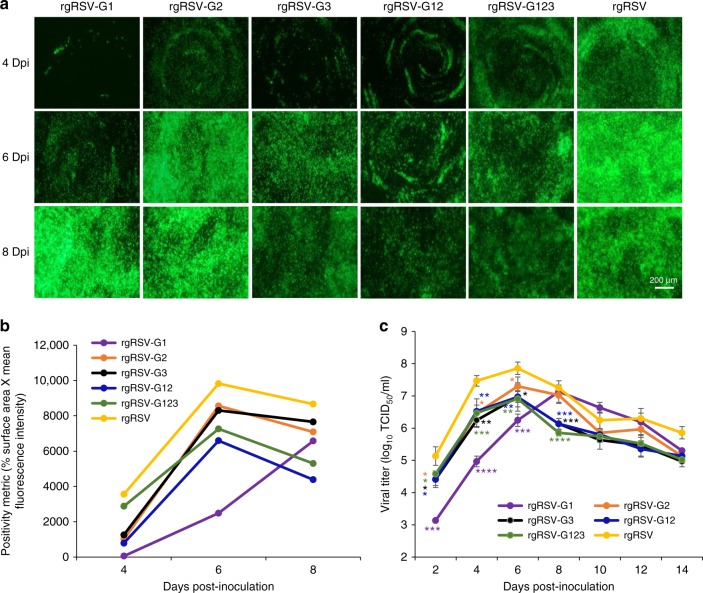
m^6^A-abrogating RSV mutants have defects in replication in HAE culture. **a** Spreading of m^6^A mutated rgRSVs in HAE culture. HAE cultures were infected by 800 TCID_50_ of each rgRSV. At the indicated time, virus spreading was monitored by fluorescence microscopy. Representative micrographs with ×4 magnification (scale bar of 200 μm) at each time point are shown. **b** Quantification of GFP signal in HAE culture. GFP signal was quantified by Image J software, and data are expressed as mean of three transwells of HAE culture, and are the representatives of three independent experiments. **c** Virus release from m^6^A mutated rgRSV-infected HAE culture. HAE cultures were infected by 800 TCID_50_ of each rgRSV. After virus inoculation, supernatants were collected every 2 days until day 14 post-inoculation. Infectious virus in supernatants was determined by TCID_50_ assay. Viral titers are the geometric mean titer (GMT) of three independent experiments ± standard deviation. The *P* value (Student’s *t*-test) for rgRSV-G1 at days 2, 4, and 6 is ***P = 0.000277, ****P = 5.15 × 10^−5^, and ***P = 0.000513, respectively; for rgRSV-G2 at days 2, 4, and 6 is *P = 0.0315, *P = 0.0213, and *P = 0.0504, respectively; for rgRSV-G3 at days 2, 4, 6, and 8 is *P = 0.0315, **P = 0.00115, *P = 0.0330, and ***P = 0.000565 respectively; for rgRSV-G12 at days 2, 4, 6, and 8 is *P = 0.0227, **P = 0.00302, **P = 0.00375, and ***P = 0.000565 respectively; for rgRSV-G123 at days 2, 4, 6, and 8 is *P = 0.0342, ***P = 0.000486, **P = 0.00682, and ****P = 6.07 × 10^−5^ respectively

We next tested the replication and spread of m^6^A-deficient rgRSVs in HAE cultures, a near in vivo model for lower airway infection. These cultures are pseudostratified and polarized, closely resembling the in vivo airway epithelium morphology and function, including mucus production and ciliary motion^47^. In brief, HAE cultures were infected with 800 TCID_50_ of each recombinant virus, and viral release and spread was monitored. As in A549 cells, m^6^A-mutated rgRSVs had a delay in viral gene expression (GFP production) and spread (Fig. 8a, b). At day 4 post-inoculation, m^6^A-mutated rgRSVs had fewer green cells compared to rgRSV. Although several rgRSV mutants gradually increased at days 6 and 8, the density of green cells remained less than for rgRSV. rgRSV-G1 was delayed in spreading but eventually spread to most susceptible cells at day 8. rgRSV-G2 had a delay at the early time point (day 4) but had wild-type level of spreading at later times. Recombinant rgRSV-G12 was the most defective virus in HAE cultures, displaying a weak GFP signal during the entire experimental period. In addition, m^6^A-mutated rgRSVs had delays in virus release in HAE culture with 1–2 log defects in virus yield (Fig. 8c). These results demonstrate that m^6^A-mutated rgRSVs were defective in replication and spread in this near in vivo lung infection model.

### m^6^A-mutated rgRSVs are defective in replication in vivo

**Fig. 9:**
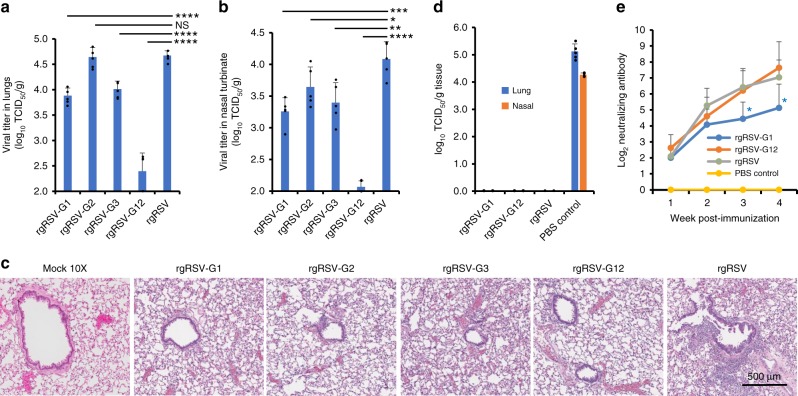
Pathogenicity and immunogenicity of m^6^A-mutated rgRSVs in cotton rats. **a** RSV titer in lungs. Four-week-old SPF cotton rats were inoculated intranasally with 2.0 × 10^5^ TCID_50_ of each rgRSV. At day 4 post-infection, the cotton rats were killed, and lungs and nasal turbinates were collected for virus titration by TCID_50_ assay. Viral titers are the geometric mean titer (GMT) of 5 animals ± standard deviation. Detection limit is 2.0 log TCID_50_/g tissue. The *P* value (Student’s *t*-test) for rgRSV-G1, rgRSV-G3, and rgRSV-G12 is ****P = 9.99 × 10^−6^, ****P = 4.06 × 10^−5^, and ****P = 7.89 × 10^−7^ respectively. **b** RSV titer in nasal turbinates. The *P* value (Student’s *t*-test) for rgRSV-G1, rgRSV-G2, rgRSV-G3, and rgRSV-G12 is ***P = 0.000740, *P = 0.0460, **P = 0.00623, and ****P = 2.88 × 10^−7^ respectively. **c** m^6^A mutated rgRSVs had less lung histopathological changes compared to rgRSV. Representative pathological changes from each group are shown. Right lung lobe of each cotton rat was fixed in 4% neutral buffered formaldehyde, embedded in paraffin, sectioned at 5 µm, and stained with hematoxylin-eosin (HE) for the examination of histological changes by light microscopy. Micrographs with ×20 magnification (scale bar of 500 μm) are shown. **d** m^6^A mutated rgRSV provides complete protection against RSV challenge. Four-week-old SPF cotton rats were inoculated intranasally with 2.0 × 10^5^ TCID_50_ of each rgRSV. At week 4 post-immunization, cotton rats were challenged with 2.0 × 10^5^ TCID_50_ rgRSV. At day 4 post-challenge, the cotton rats were killed, and lungs and nasal turbinates were collected for virus titration by TCID_50_ assay. Viral titers are the geometric mean titer (GMT) of 5 animals ± standard deviation. The detection limit is 2.0 log TCID_50_/g tissue. **e** rgRSV induced a high level of neutralizing antibody. Blood samples were collected from each rat weekly by retro-orbital bleeding. The RSV-neutralizing antibody titer was determined using a plaque reduction neutralization assay, as described in Materials and Methods. The *P* value (Student’s *t*-test) for rgRSV-G1 at week 3 and 4 is *P = 0.0166 and *P = 0.0490, respectively

We tested replication and pathogenesis of four m^6^A-mutated rgRSV mutants, rgRSV-G1, G2, G3, and G12, in cotton rats. The parental rgRSV replicated efficiently in the lungs (Fig. 9a) and nasal turbinates (Fig. 9b) with average viral titers of 4.70 ± 0.10 log_10_ TCID_50_/g and 4.10 ± 0.10 log_10_ TCID_50_/g, respectively. Mutant rgRSV-G1 had a 7-fold reduction in replication in nasal turbinate and lung titers, respectively (*P* < 0.05). Mutant rgRSV-G2 had no significant reduction in replication in lung (*P* > 0.05) but 3-fold reduction in nasal turbinate (*P* < 0.05). Mutant rgRSV-G12 had the most dramatic defect in replication, with reductions of 100- and 200-fold in viral titer in nasal turbinate and lung, respectively. It should be noted that 4 out of 5 cotton rats had below detection limit level of RSV replication in the nasal turbinate and 3 out of 5 cotton rats had below detection limit level of RSV replication in lungs, suggesting that rgRSV-G12 is highly attenuated in vivo. The rgRSV-G3 had 5-fold reduction in replication in nasal turbinate and lung (*P* < 0.05). Histologic examination showed that rgRSV caused moderate pulmonary histopathological changes, including interstitial pneumonia and peribronchial lymphoplasmocytic infiltrates (Fig. 9c). In contrast, m^6^A-deficient rgRSV mutants only had mild and less pulmonary histopathological changes compared to rgRSV (Fig. 9c). These results showed that m^6^A-deficient rgRSV mutants had significant reductions in viral replication in both the upper and lower respiratory tracts in cotton rats and were less pathogenic compared to rgRSV. These results indicate that viral m^6^A upregulates viral replication and pathogenesis in vivo.

### m^6^A-mutated rgRSVs are highly immunogenic in cotton rats

To determine whether defects in viral m^6^A methylation impair the immunogenicity of the virus, we evaluated the protection efficacy of a partially attenuated (rgRSV-G1) and highly attenuated (rgRSV-G12) virus in cotton rats. Cotton rats immunized with parental rgRSV or m^6^A-mutated rgRSVs did not have any detectable infectious virus in either the nasal turbinate or lung tissue after challenge with rgRSV (Fig. 9d). In contrast, unvaccinated challenged controls had average titers of 5.12 ± 0.28 and 4.27 ± 0.07 log_10_ PFU/g in the lung and nasal turbinate, respectively (Fig. 9d). These results demonstrate that immunization with the rgRSV-G1 and G12 provided complete protection from challenge with rgRSV. Lung histology showed that unvaccinated challenged controls had moderate histologic lesions (Supplementary Fig. 15). However, the vaccinated challenged groups had only mild lesions in lungs. In addition, no enhanced lung damage was observed (Supplementary Fig. 15). The most attenuated m^6^A-deficient virus (rgRSV-G12) triggered similar levels of neutralizing antibody compared to rgRSV (*P* > 0.05) (Fig. 9e). The rgRSV-G1 had a lower antibody titer at week 3 and 4 compared to rgRSV (Fig. 9e). These results demonstrate that m^6^A-mutated rgRSV retained high immunogenicity and provided complete protection against RSV infection in cotton rats.

### m^6^A-mutated rgRSVs are less dependent on host m^6^A machinery

**Fig. 10:**
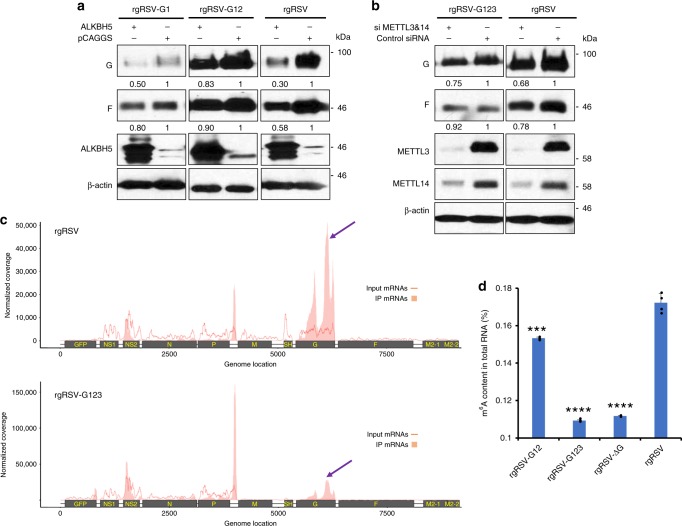
The attenuated phenotype of m^6^A mutated rgRSVs is m^6^A-related. **a** rgRSV-G1 and -G12 were less dependent on the m^6^A eraser protein. A549 cells were transfected with a plasmid encoding ALKBH5. At 36 h post-transfection, cells were infected with each rgRSV at an MOI of 0.5. At 18 h post-infection, cell lysates were harvested for western blot analysis. Western blot images are the representatives of three experiments. **b** rgRSV-G123 expression was less dependent on m^6^A writer protein. A549 cells were transfected with control siRNA or siRNA targeting METTL3 and METTL14. At 36 h post-transfection, cells were infected with each rgRSV at an MOI of 0.5. At 18 h post-infection, cell lysates were harvested for western blot analysis. The density of western blot was quantified by Image J software, and the ratio of the protein bands was calculated. Images are the representatives of three experiments. **c** Distribution of m^6^A peaks on the RSV mRNAs from A549 cells infected by rgRSV and rgRSV-G123. Confluent A549 cells were infected by each m^6^A-mutated rgRSV at an MOI of 1.0, cell lysates were harvested at 36 h post-infection. Total RNAs were extracted from cell lysates, and were enriched for mRNA by binding to oligo dT, and subjected to m^6^A-seq. The distribution of m^6^A-immunoprecipitated (IP) reads were mapped to the RSV mRNAs (pink block). The baseline distributions for mRNAs from input sample are shown as a pink line. Data presented are the mean coverage from two independent virus-infected A549 cell samples (*n* *=* 2). Red arrow indicates the m^6^A enrichment in G mRNA. **d** Virion RNA of m^6^A-mutated rgRSVs is defective in binding to anti-m^6^A antibody. Virion RNA was extracted from highly purified RSV virions. Antigenome was quantified by real-time RT-PCR. Each amount of antigenome was bound to strip wells using a RNA high binding solution, and m^6^A was detected using a specific capture anti-m^6^A antibody and then quantified colorimetrically by reading the absorbance in a microplate spectrophotometer at a wavelength of 450 nm. A standard curve was generated using known m^6^A methylated RNA (range from 0.02 to 1 ng of m^6^A) as a positive control. The m^6^A content was calculated from each RNA samples. Data are averages of four independent experiments. The *P* value (Student’s *t*-test) for rgRSV-G12, rgRSV-G123, and rgRSV-ΔG is ***P = 0.000325, ****P = 3.09 × 10^−7^, and ****P = 3.74 × 10^−7^, respectively

If the attenuated phenotype of m^6^A-mutated rgRSVs is indeed m^6^A-dependent, alteration of host m^6^A machinery would have no or less of an impact on replication and gene expression. To address this question, we tested the replication of rgRSV-G1 and G12 in A549 cells overexpressing ALKBH5 which is an m^6^A eraser protein. Overexpression of ALKBH5 led to 70% and 42% reduction in RSV G and F protein synthesis in rgRSV-infected cells compared to vector control cells (Fig. 10a). We also observed a reduction in replication and protein expression of the m^6^A-mutated rgRSVs in ALKBH5 overexpressing cells, but the level of reduction was much less compared to the parental rgRSV. For example, only 17% and 10% reduction in RSV G and F protein synthesis was observed for rgRSV-G12, and 50% and 20% reduction in G and F protein was observed for rgRSV-G1, respectively. We also tested the replication of rgRSV-G123 in m^6^A writer protein-depleted A549 cells. Knockdown of MELL3 and METTL14 led to 32 and 22% reduction in RSV G and F protein in rgRSV-infected A549 cells whereas only 25% and 8% reduction in G and F in rgRSV-G123-infected A549 cells (Fig. 10b). Thus, these results showed that replication and gene expression of m^6^A-mutated rgRSVs were less dependent on host m^6^A machinery, suggesting that the attenuated phenotype of these mutants is likely due to the deficiency in m^6^A methylation of the viral RNA.

### m^6^A-mutated rgRSVs are deficient in m^6^A enrichment

To determine whether m^6^A sites are indeed missing from the G gene, A549 cells were infected by each m^6^A-mutated rgRSV, and polyadenylated mRNAs were isolated and subjected to m^6^A-seq. As shown in Fig. 10c and Supplementary Fig. 16, the enrichment of m^6^A in the G mRNA of each m^6^A-mutated rgRSV significantly decreased compared to the G mRNA from the parental rgRSV, confirming that m^6^A methylation in the G mRNA has indeed been significantly reduced. Our mutagenesis targeted the positive-sense RNA which includes both antigenome and G mRNA. To determine whether antigenome is also defective in m^6^A methylation, virion RNA was extracted from highly purified virions of rgRSV, rgRSV-G12, rgRSV-G123, and rgRSV-ΔG (rgRSV with deletion of entire G gene), and m^6^A content in total RNA was measured. As shown in Fig.10d, m^6^A content in virion RNA of rgRSV-G12, rgRSV-G123, and rgRSV-ΔG was significantly less compared to those in rgRSV (*P* < 0.05). In addition, there was no significant difference in m^6^A content between rgRSV-G123, and rgRSV-ΔG (*P* > 0.05). Collectively, these results demonstrate that m^6^A-mutated rgRSVs indeed have defects in m^6^A methylation.

## Discussion

The biological function of m^6^A methylation in viral RNAs has remained uncertain since its discovery 40 years ago. Recent studies have shown that viral m^6^A methylation can have pro-viral or antiviral functions dependent on the specific virus species and cell type. Here, we showed that m^6^A modification positively regulates each step in the RSV replication cycle ranging from genome replication, mRNA transcription, and viral protein synthesis, to progeny infectious particle production. We demonstrated that m^6^A regulates RSV replication and pathogenesis in an animal model. Furthermore, we provide evidence that m^6^A could be a target for the development of live attenuated vaccine candidates.

The m^6^A methylation of RNAs is modulated by writers, erasers, and readers in host cells. It should be noted that m^6^A methylation and its reader proteins may play distinct roles in a virus life cycle. In this study, we showed that overexpression of both m^6^A reader and writer proteins positively regulated RSV replication while knockdown inhibited RSV gene expression and replication. The opposite was true for eraser proteins: overexpression decreased RSV gene expression and replication whereas knockdown increased them. In case of influenza virus, m^6^A methylation enhanced viral replication as did the reader, YTHDF2, but not YTHDF1 or 3^33^. We found that overexpression of m^6^A writer proteins led to an increased level of m^6^A in viral RNA. The impact of m^6^A-related proteins on RSV replication may result from three aspects: a change in m^6^A levels in viral RNA which directly affects viral replication, a change in host gene expression which indirectly affect viral replication, or both. However, m^6^A writer and m^6^A reader proteins have been found to negatively regulate HCV production^38^, opposite to RSV and influenza virus. In addition, the role of m^6^A reader proteins in the HIV life cycle is controversial^30–32^. Thus, m^6^A readers have distinct effects on the life cycles of different viruses, as they are multifunctional and play many important biological roles ranging from RNA stability, decay, and transport, to protein translation.

Our m^6^A-seq also found that the viral G mRNA has the most abundant m^6^A peaks among the 10 RSV mRNAs in both HeLa and A549 cells. In addition, the strongest m^6^A peaks in both the genome and the antigenome are located in the *G* gene region. Another interesting finding was that the positions of the m^6^A modifications in the genome and antigenome largely overlapped despite the fact that the sequence of the antigenome is complementary to the genome. However, it is unclear why antigenome and genome are m^6^A methylated because both of them are completely encapsidated by viral N protein. It was shown that RSV RNA synthesis occurs in cytoplasmic inclusion bodies^48^. In fact, m^6^A-related proteins are partially co-localized with viral N protein (Fig. 6a, b), suggesting that host m^6^A machinery may interact with RSV ribonucleoprotein (RNP) complex. It is possible that m^6^A methylation occurs prior to N encapsidation, or m^6^A methylation and encapsidation can occur concurrently.

Since G mRNA has the strongest m^6^A enrichment, we searched the three peaks in the G sequence for m^6^A motifs, identifying a total of 18 putative m^6^A sites. It is known that the G gene is the most genetically diverse RSV gene. However, bioinformatics analysis of 100 RSV strains (Supplementary Fig. 17) found that those 18 m^6^A sites are highly conserved in the *G* gene, suggesting that m^6^A sites in the *G* gene may provide an evolutionary advantage for virus infection, replication, and spreading. Mutations in these three m^6^A peaks in the G mRNA showed that peaks 1 and 3 play a major role in regulating RSV replication whereas peak 2 plays a minor role, as recombinant rgRSV mutants in peak 1 and 3 (rgRSV-G1 and G3) had greater deficits in replication compared to mutants in peak 2 (rgRSV-G2).

The G protein is primarily responsible for the attachment of RSV to host cells and plays a role in modulating innate immune responses^47,49^. Although it is not essential for the production of infectious RSV, RSV G is necessary for full infectivity^50,51^. The G protein also plays an important role in the assembly of filamentous virions^52^. It is likely that the abundant m^6^A modifications of the G mRNA enhances its stability, enabling more translation, which may lead to insertion of more G protein into virions and enhanced viral assembly and production of infectious virions. However, a portion of the G protein produced in a cell is released in a soluble form that affects leukocyte migration^53^. Enhanced G protein expression could enhance the production of soluble G protein, thereby affecting the immune response to RSV. It is also possible that m^6^A modification of viral RNAs facilitate virus escape from the surveillance of host innate immunity to allow for efficient gene expression and virus replication. In this study, we also found that rgRSVs carrying mutations in the m^6^A sites in G not only decreased G protein expression but also reduced expression of other viral proteins (such as N and F proteins). Since RSV replication and transcription require ongoing protein synthesis, a reduction in viral G protein expression will affect the second round of viral infection, which results in a reduction of overall replication and transcription, and expression of all viral proteins.

Importantly, we found that viral m^6^A also modulates viral replication and pathogenesis in vivo. However, the degree of attenuation in cell culture did not always match that in vivo. For example, rgRSV-G1 and G12 had similar levels of attenuation in immortalized cells (A549 and HeLa cells). In cotton rats, rgRSV-G1 replication was 7-fold reduced in the lung and nasal turbinate, respectively, whereas rgRSV-G12 had more than 100-fold reductions. Recombinant rgRSV-G2 was only mildly attenuated in cell culture. This recombinant had similar level of replication in lungs (*P* > 0.05), and only had threefold reduction in nasal turbinates (*P* < 0.05). Therefore, it appears that m^6^A sites in peaks 1 and 2 contributed synergistically to the highly attenuated phenotype of rgRSV-G12 in vivo. The phenotype of these mutants in HAE culture seems to correlate better with the phenotype in cotton rats than in HeLa and A549 cells. Thus, HAE culture may be better system to predict virus replication in vivo. Also, m^6^A-mutated rgRSVs had significantly less histopathology compared to the parental rgRSV. These results demonstrate that m^6^A not only modulates the virus life cycle in vitro but also regulates viral replication and pathogenesis in vivo.

Recent  studies in vesicular stomatitis virus (VSV) found that codon usage also affected genome replication and mRNA transcription in a minigenome assay^54^. Specifically, purine/pyrimidine content in RNA template modulates the stability of the polymerase complex, which results in alteration of the activity of viral RNA synthesis^54^. In this study, we designed mutations in predicted m^6^A sites to avoid as much as possible alterations to the predicted mRNA secondary structure and to avoid changes in the efficiency of gene expression of the new codon relative to the original codon. We also confirmed the loss of m^6^A in the predicted region of the G mRNA by m^6^A sequencing and tested the functional consequences of reducing the m^6^A modifications. Functional loss of m^6^A modifications was examined by comparing replication of the mutant rgRSV in A549 cells overexpressing or depleted of m^6^A-related proteins. The m^6^A-mutated rgRSVs (G1, G12, and G123) were much less dependent on host m^6^A enzyme compared to the parental rgRSV, confirming that the attenuated phenotype of m^6^A-mutated rgRSVs is due to the reduction of m^6^A sites in G mRNA. Removal of m^6^A sites in the mRNA also removes them from the antigenome, but not from other viral mRNAs, other locations in the antigenome, or sites in the genome. Therefore, rgRSVs lacking particular m^6^A peaks in the G gene would be partially but not fully independent of host m^6^A enzymes.

A potentially important application of this study is in the rational design of live attenuated RSV vaccine candidates by inhibiting m^6^A addition to the mRNA and antigenome, or perhaps the viral genome. Currently, there is no FDA-approved vaccine for RSV despite the fact that it was first isolated in 1953. A live attenuated vaccine, similar to the effective vaccines for the related measles and mumps viruses, would seem to be one of the most promising methods for protection from RSV disease. However, it has been a challenge to strike the right balance between attenuation and immunogenicity^42^.

Although mutations in individual m^6^A peaks in the G mRNA were not sufficient to achieve complete attenuation of RSV replication in vivo, the combination of m^6^A mutations in peaks 1 and 2 resulted in a recombinant virus that was sufficiently attenuated both in vitro and in vivo. Importantly, cotton rats vaccinated with rgRSV-G12 had similar neutralizing antibody response levels compared to parental rgRSV and were completely protected from rgRSV challenge. In addition, no enhanced lung damage was observed. Thus, rgRSV-G12 may be a good live attenuated vaccine candidate for RSV. This study demonstrates that inhibition of m^6^A methylation may be a novel method for rationally designing live attenuated vaccines. These m^6^A-mutated rgRSVs would also provide invaluable tools to understand the roles of m^6^A modification in the innate immune response. In fact, it has been shown that internal m^6^A modification of in vitro synthesized RNAs prevents recognition of the RNA by the host pattern recognition receptors TLR3 and RIG-I^55^.

This study also provides a novel approach for enhancing viral titers in cell culture, an important consideration in the production of live attenuated vaccines. Attenuated viruses typically grow to lower titers than wild-type virus. In the case of RSV, a relatively large dose of vaccine candidate is required to induce a protective immune response in humans, making vaccine production expensive. One strategy might be to produce live attenuated vaccines in cells overexpressing one or more m^6^A reader or writer proteins, since overexpression of these host m^6^A machinery components enhance virus yield at least 10-fold. Such a boost in the production of a vaccine should greatly enhance its economic feasibility. Unfortunately, this approach would not be useful for m^6^A mutant attenuated viruses, such as the ones described in this report. It should be useful for viruses attenuated by any other method.

In summary, we mapped the internal m^6^A modifications in RSV RNAs and showed that m^6^A enhances RSV replication, gene expression, and virus production. In addition, we provide evidence that m^6^A upregulates RSV pathogenesis and virulence in vivo. These findings highlight viral m^6^A machinery as a possible novel target for rational design of live attenuated vaccines, for enhanced production of live attenuated vaccines, and for broad-spectrum antiviral drug discovery.

## Method

### Ethics statement

The animal study was conducted in strict accordance with USDA regulations and the recommendations in the Guide for the Care and Use of Laboratory Animals of the National Research Council and was approved by The Ohio State University Institutional Animal Care and Use Committee (IACUC; animal protocol no. 2009A0221). The animals were housed within the University Laboratory Animal Resources (ULAR) facilities of The Ohio State University according to the guidelines of the Institutional Animal Care and Use Committee (IACUC). The animal care facilities at The Ohio State University are AAALAC accredited. Every effort was made to minimize potential distress, pain, or discomfort to the animals throughout all experiments.

### Cell lines

HeLa (ATCC CCL-2), A549 (ATCC CCL-185), Vero (ATCC CRL-CCL81), and HEp-2 (ATCC CCL-23) cell lines were purchased from the American Type Culture Collection (Manassas, VA) and were grown in Dulbecco’s modified Eagle’s medium (DMEM; Life Technologies) supplemented with 10% FBS. HeLa cells overexpressing the empty vector (pPB-CAG), YTHDF1, YTHDF2, or YTHDF3 were maintained in DMEM, 10% FBS and 1 µg/ml of puromycin every passage to select for YTHDF1-3 overexpressing cells. Primary, well-differentiated human airway epithelial (HAE) cultures were grown on collagen coated Transwell inserts (Corning Incorporated, Corning, NY) at an air-liquid interface, as previously described^47^. Upon reaching confluency and forming tight junctions, the apical medium was removed and cultures were maintained at the air–liquid interface for 4–6 weeks to generate well-differentiated, polarized cultures. All cell lines used in this study were free of mycoplasma, as confirmed by the LookOut Mycoplasma PCR Detection Kit (Sigma).

### Virus stocks and purification

Recombinant RSV containing a green fluorescence protein (*GFP*) gene between the leader sequence and *NS1* gene (rgRSV)^47^ was propagated and titered in HeLa cells or A549 cells. To prepare purified rgRSV, 20 T150 flasks of HeLa cells or A549 cells were infected by rgRSV at an MOI of 0.1, and cell culture supernatants harvested at 48 or 72 h post-infection were clarified by centrifugation at 10,000 × *g* for 30 min. Virus was concentrated through a 35% (wt/vol) sucrose cushion by centrifugation at 30,000 × *g* for 2 h at 4 °C in a Ty 50.2 rotor (Beckman). The pellet was resuspended in DMEM with 10% trehalose and further purified through a sucrose gradient (20–55%) by centrifugation at 35,000 × *g* for 2 h at 4 °C in an SW55 rotor (Beckman). The final pellet was resuspended in 0.5 ml of DMEM with 10% trehalose.

### m^6^A-seq

High-throughput sequencing of the RSV and host methylome was carried out using m^6^A-seq (MeRIP-seq) as described previously^20^. For m^6^A-seq of the rgRSV genome and antigenome, RNAs were extracted from purified rgRSV virions and purified with the RiboMinus Eukaryote System v2 kit (Thermo Fisher). For m^6^A-seq of host transcripts, total RNAs were extracted from mock or rgRSV-infected HeLa or A549 cells and polyadenylated RNAs were isolated using Dynabeads mRNA DIRECT Purification kit (Thermo Fisher). Purified RNAs were sonicated with Bioruptor Pico (Diagenode) with 30s ON 30s OFF for 30 cycles, mixed with 1 µl of affinity purified anti-m^6^A monoclonal antibody (NEB) in IPP buffer (150 mM NaCl, 0.1% NP-40, 10 mM Tris-HCl, pH 7.4) and incubated for 2 h at 4°C. Enriched mRNA fragments were purified with RNA Clean & Concentrator kit (Zymo) and used for library generation with TruSeq Stranded mRNA Library Prep kit (Illumina). Sequencing was carried out on Illumina HiSeq 4000 according to the manufacturer’s instructions. Two replicates of RNA samples from virions, virus-infected cells, and mock-infected cells were subjected to m^6^A-seq. For data analysis, after removing the adapter sequences, the reads were mapped to the human genome (hg38) and rgRSV genome and antigenome by using Hisat2^56^ with peak calling as described^57^. Metagene analysis was performed by R package Guitar^58^. Differential methylation analysis was performed with count based negative binomial model implemented in QNB test^46^.

### Quantification of RSV RNA m^6^A level using LC-MS/MS

RSV RNA (250 mg) was extracted from highly purified rgRSV virions using an RNeasy Mini kit (Qiagen) and purified twice with RiboMinus Eukaryote System v2 kit (Thermo Fisher). To examine the purity of virion RNA, oligo d(T) was used for reverse transcription, followed by qPCR for quantification for β-actin and viral N and G mRNAs. Virion RNA which was free of contamination of host RNA and viral mRNAs was used for liquid chromatography-mass spectrometry (LC-MS/MS), m^6^A antibody pulldown assay, and m^6^A-seq. Purified RNA was digested and subjected to a quantitative analysis of the m^6^A level using LC-MS/MS as previously described^7^.

### Quantification of RSV RNA m^6^A level using anti-m^6^A antibody

A549 cells in T150 flasks were transfected with 10 µg of plasmids encoding METTL3 and METTL14, ALKBH5, or vector pCAGGS. For siRNA transfection, A549 cells in T75 flasks were transfected with 150 pmole of siRNA targeting METTL3 and METTL14, ALKBH5, or control siRNA. At 24 h post-transfection, the transfected cells were infected with rgRSV at an MOI of 0.1. At 42 h post-infection, cell culture supernatants (containing RSV particles) were harvested. RSV particles were pelleted and purified through ultracentrifugation. Virion RNA was extracted from highly purified RSV virions. Antigenome was quantified by real-time RT-PCR. Each amount of antigenome was bound to strip wells using a RNA high binding solution, and m^6^A was detected using a specific capture anti-m^6^A antibody (Abcam, ab185912) and then quantified colorimetrically by reading the absorbance in a microplate spectrophotometer at a wavelength of 450 nm. A standard curve was generated using known m^6^A methylated RNA (range from 0.02 to 1 ng of m^6^A) as a positive control. The m^6^A content was calculated from each RNA samples based on their OD450 values. The percent of changes was calculated by dividing m^6^A contents in viral RNA from the treated group by those from the control group.

### Host cell gene differential expression analysis

Host cell differential gene expression was analyzed by R package DESeq2^58^ using wald-test. The significantly differentially expressed genes were reported at adjusted *P* value cutoff of 0.05.

### GO analysis

Gene Ontogeny (GO) analysis was performed using the R package cluster Profiler^58^. Specifically, enrichKEGG function was called to analyze for enriched pathway and enrichMap function was called to generate network plot of enriched pathway.

### Plasmids and site-directed mutagenesis

The plasmid vector was used to overexpress the readers (YTHDF1-3), writers (METTL3, METTL14), and erasers (FTO, ALKBH5) as described previously^32^. Plasmid (RW30) encoding the full-length antigenomic cDNA of RSV strain A2 with GFP inserted between the leader and the NS1 gene, and support plasmids expressing RSV A2 strain N protein (pTM1-N), P protein (pTM1-P), L protein (pTM1-L), and M2-1 protein (pTM1-M2-1) were generously provided by Dr. P.L. Collins, NIAID, Bethesda, MD. Mutations to the potential m^6^A sites in G gene were introduced into the RW30 plasmids using QuikChange site-directed mutagenesis kit (Stratagene, La Jolla, CA). The m^6^A peaks in the G gene are clustered in three regions, 392–467 nt, 567–660 nt, and 716–795 nt. Since it is known that m^6^A modified sites in RNA contain the conserved Pu [G > A]m^6^AC[A/C/U] motif (Pu represents purine)^1^, we searched for this motif in these three regions in G mRNA and identified 6, 7, and 4 potential m^6^A sites in regions G1, G2, and G3 respectively (Supplementary Fig. 13). The potential m^6^A sites mutants in G1 peak include 394-AGm^6^ACC-400; 401-AAm^6^ACA-407; 418-AAm^6^ACA-424; 444-AAm^6^ACA-450; 455-AAm^6^ACA-461; 459-AAm^6^ACC-465; mutants in G2 peak include 569-AAm^6^ACA-575; 576-AAm^6^ACC-582; 589-AAm^6^ACC-595; 612-AAm^6^ACC-618; 625-AGm^6^ACA-631; 645-AAm^6^ACC-651; 652-AAm^6^ACC-658; and mutants in G3 peak include 718-AAm^6^ACA-724; 722-AAm^6^ACA-728; 768-GAm^6^ACT-774; 787-AAm^6^ACC-793 (Supplementary Fig. 13). The A or C within the consensus m^6^A sites was mutated to a T or G in these sites without changing the encoded amino acid. Mutant G12 combined the mutations from G1 and G2. Mutant G123 was a combined the mutations from G1, G2, and G3. In addition, M-fold and Genscript software were used to predict that these mutations did not alter RNA secondary structure or codon usage. All plasmids and mutations were confirmed by DNA sequencing.

### siRNA and siRNA transfection

siRNAs against METTL3, METTL14, FTO, ALKBH5, YTHDF1, YTHDF2, YTHDF3, or non-targeting AllStars negative control siRNA were purchased from Qiagen (Valencia, CA, sequences listed in Supplementary Table 7). All siRNA transfections were performed using the Lipofectamine 3000 transfection reagent (Thermo-Fisher) according to the manufacturer’s instructions.

### Antibodies and Western blotting

The antibodies used in this study were anti-YTHDF1 (1:1000, Proteintech, Rosemont, IL), anti-YTHDF2 (1:1000, Abcam, Cambridge, MA), anti-YTHDF3 (1:1000, Abcam), anti-METTL3 (1:1000, Proteintech), anti-METTL14 (1:1000, Proteintech), anti-ALKBH5 (1:1000, Sigma-Aldrich, St. Louis, MO), anti-FTO (1:1000, Abcam), and anti-RSV serum (1:400, Virostat, Westbrook, ME), F (1:3000, Abcam), anti-FLAG (1:3000, Sigma-Aldrich), anti-Actin (1:5000, Proteintech), and anti-Tubulin (1:5000, Abcam). Cells were harvested and lysed in RIPA buffer (Abcam) supplemented with protease inhibitor cocktail (Sigma-Aldrich). Western blotting was performed as described. Tubulin or actin was used as a loading control.

### Immunofluorescence analysis and confocal microscopy

Mock or rgRSV-infected cells were fixed in acetone and methanol at the ratio of 1:1 for 30 min, and blocked with 10% goat serum. Slides were stained with all primary antibodies (1:100), washed three times with PBST, and stained with conjugated Alexa Fluor secondary antibodies Alexa Fluor 488/594 (1:300, Thermo-Fisher), and mounted with SlowFade™ Diamond Antifade Mountant with DAPI (Thermo-Fisher). Imaging was performed on an Olympus FV 1000 confocal microscopy system at The Ohio State University Campus Microscopy & Imaging Facility.

### Real-time RT-PCR

RSV genome, antigenome, and mRNA were quantified by real-time RT-PCR. HeLa or A549 cells were infected with rgRSV or an rgRSV mutant at an MOI of 0.1. At 12, 18, and 24 post-infection, total RNA was isolated from cells using TRIzol (Life Technologies). Viral genome or antigenome copies were quantified by real-time RT-PCR using two primers specifically targeting the RSV leader sequence and GFP gene (Supplementary Table 8). Poly (A)-containing viral mRNAs were isolated from total RNA using a Dynabead mRNA isolation kit (Life Technologies) according to the manufacturer’s recommendations. Using the viral mRNAs as the template, the NS1 and G mRNA copies were quantified by real-time RT-PCR using two primers targeting the viral *NS1* and *G* genes, respectively.

### RNA-immunoprecipitation

The RNA-immunoprecipitation (RIP) assay was performed as described previously^38^. In brief, HeLa cells were infected with rgRSV at MOI of 1.0 and cell extracts were harvested in polysome lysis buffer after 36 h post-infection. RNP complexes were immunoprecipitated with anti-HA antibody conjugated to magnetic beads (Sigma) or anti-YTHDF2 antibody overnight at 4 °C, and washed five times with ice-cold NT2 buffer. For the RIP with anti-YTHDF2 antibody, additional secondary antibody was added. After the final wash, 10% of the beads were used for immunoblotting and the remaining 90% were used for RNA extraction using TRIzol (ThermoFisher).

### Recovery of RSV from the full-length cDNA clones

rgRSV mutants were rescued from the full-length cDNA of the RSV A2 strain^59^. HEp-2 cells were infected with MVA-T7 at an MOI of 10, then transfected with 1.2 µg of plasmid RW30 or RW30 mutant, 0.4 µg of pTM1-N, 0.2 µg of pTM1-P, 0.1 µg of pTM1-M2-1, and 0.1 µg of pTM1-L using the Lipofectamine 3000 reagent (Life Technologies). At day 4 post-transfection, the cells were harvested using scrapers and were co-cultured with new flask of HEp-2 cells at 50–60% confluence. When an extensive cytopathic effect (CPE) was observed, the cells were subjected to three freeze-thaw cycles, followed by centrifugation at 4000 × *g* for 10 min. The supernatant was subsequently used to infect new HEp-2 cells. The successful recovery of the rgRSV was confirmed by the presence of green fluorescent cells, followed by RT-PCR and sequencing. Recombinants rgRSV carrying mutations in m^6^A sites were designated as rgRSV-G1, G2, G3, G12, and G123.

### RT-PCR and sequencing

All plasmids, viral mutants and stocks, and virus isolates from the nasal turbinates and lungs of cotton rats were sequenced to confirm virus identity. Viral RNA was extracted from 100 µl of each recombinant virus using an RNeasy minikit (Qiagen, Valencia, CA). A 1.5-kb DNA fragment spanning the RSV *G* gene was amplified by RT-PCR. The PCR products were purified and sequenced using a sequencing primer at The Ohio State University Plant Microbe Genetics Facility to confirm the presence of the designed mutations.

### Viral replication kinetics

Confluent HeLa or A549 cells in 6-well-plate were infected with wild-type rgRSV or mutant rgRSV at an MOI of 0.1. After 1 h of adsorption, the inoculum was removed and the cells were washed three times with DMEM. Fresh DMEM (supplemented with 2% FBS) was added, and the infected cells were incubated at 37 °C. At different time points post-inoculation, the supernatant and cells were harvested by three freeze-thaw cycles, followed by centrifugation at 1500 × *g* at room temperature for 15 min. The virus titer was determined by TCID_50_ assay in HEp-2 cells^47^.

### Genetic stability of rgRSV mutants in cell culture

Confluent Vero cells in T25 flasks were infected with each rgRSV mutant at an MOI of 0.1. At day 3 post-inoculation, the cell culture supernatant was harvested and used for the next passage in Vero cells. Using this method, each rgRSV mutant was repeatedly passaged 15 times in Vero cells. At each passage, the *G* gene was amplified by RT-PCR and sequenced. At passage 15, the entire genome of each recombinant virus was amplified by RT-PCR and sequenced.

### Replication and pathogenesis of rgRSV in cotton rats

Thirty 6-week-old specific-pathogen-free (SPF) male cotton rats (Envigo, Indianapolis, IN) were randomly divided into 6 groups (5 cotton rats per group). Prior to virus inoculation, the cotton rats were anesthetized with isoflurane. The cotton rats in group 1 were inoculated with 2.0 × 10^5^ TCID_50_ of parental rgRSV and served as positive controls. The cotton rats in groups 2–5 were inoculated with 2.0 × 10^5^ TCID_50_ of four m^6^A deficient rgRSV mutants, rgRSV-G1, G2, G3, and G12. Each cotton rat was inoculated intranasally with a volume of 100 μl. At day 4 post-infection, the cotton rats were killed via carbon dioxide inhalation. The left lung and nasal turbinates were collected for virus titration and the right lung was collected for histological analysis.

### Immunogenicity of rgRSV in cotton rats

For the immunogenicity study, twenty 6-week-old female cotton rats (Envigo) were randomly divided into five groups (5 cotton rats per group). Cotton rats in groups 1, 2, and 3 were intranasally inoculated with 2.0 × 10^5^ TCID_50_ of two m^6^A deficient rgRSV mutants (rgRSV-G1 and G12) and rgRSV, respectively. Cotton rats in groups 4 were mock-infected with DMEM and served as unvaccinated challenged control. After immunization, the cotton rats were evaluated daily for any possible abnormal reaction and blood samples were collected from each cotton rat weekly by facial vein retro-orbital plexus sampling, and serum was used for detection of neutralizing antibodies. At 4 weeks post-immunization, the cotton rats in groups 2–5 were challenged with 2.0 × 10^5^ TCID_50_ of parental rgRSV via intranasal route, and evaluated twice daily for the presence of any clinical symptoms. At 4 days post-challenge, all cotton rats were euthanized by CO_2_ asphyxiation, and their lungs and nasal turbinates were collected for virus titration. The immunogenicity of rgRSV mutants was assessed based on their ability to trigger neutralizing antibody, the ability to prevent rgRSV replication in lungs and nose, and the ability to protect lung from pathological changes.

### Pulmonary histology

After killing, the right lung of each animal was removed, inflated, and fixed with 4% neutral buffered formaldehyde. Fixed tissues were embedded in paraffin and a microtome used to generate 5 μm sections. Slides were then stained with hematoxylin-eosin (H&E) for the examination of histological changes by light microscopy. Histopathological changes were evaluated based on the extent of interstitial inflammation, edema, and peribronchiolar inflammation.

### Determination of viral titer in lung and nasal turbinate

The nasal turbinate and the left lung from each cotton rat were removed, weighed, and homogenized in either 3 ml or 2 ml of DMEM. The lung was homogenized using a Precellys 24 tissue homogenizer (Bertin, MD) by following the manufacturer’s recommendations. The nasal turbinates were homogenized by hand with a 15-mL capacity PYREX® homogenizer (Corning, NY). The presence of infectious virus was determined by TCID_50_ assay in HEp-2 cells.

### Determination of RSV-neutralizing antibody

RSV-specific neutralizing antibody titers were determined using a plaque reduction neutralization assay. In brief, cotton rat sera were collected by retro-orbital plexus sampling weekly until challenge. The serum samples were heat inactivated at 56°C for 30 min. Twofold dilutions of the serum samples were mixed with an equal volume of DMEM containing ~50 TCID_50_/well rgRSV in a 96-well plate, and the plate was incubated at room temperature for 1 h with constant rotation. The mixtures were then transferred to confluent HEp-2 cells in a 96-well plate in triplicate. After 1 h of incubation at 37°C, the virus-serum mixtures were removed and the cells were overlaid with 0.75% methylcellulose in overlay media (1 × MEM, 2% FBS, Sodium bicarbonate, 25 mM HEPES, 1% l-Glutamine, 1% Pen Strep) and incubated for another 3 days before counting the fluorescent foci. The numbers of foci at each serum dilution were plotted and the 50% plaque reduction titer was used as the RSV-specific neutralizing antibody titer.

### Statistical analysis

Quantitative analysis was performed by either densitometric scanning of autoradiographs or by using a phosphorimager (Typhoon; GE Healthcare, Piscataway, NJ), ImageQuant TL software (GE Healthcare, Piscataway, NJ), and Image J (NIH, Bethesda, MD). Statistical analysis was performed by one-way multiple comparisons using SPSS (version 8.0) statistical analysis software (SPSS Inc., Chicago, IL) or Student’s *t-*test. A *P* value of <0.05 was considered statistically significant.

### Reporting summary

Further information on research design is available in the Nature Research Reporting Summary linked to this article.

## Supplementary information

Supplementary Information

Reporting Summary

Description of Additional Supplementary Files

Supplementary Data 1

Supplementary Data 2

Supplementary Data 3

Supplementary Data 4

## Data Availability

The authors declare that the data supporting the findings of this study are available with the article and its Supplementary Information files, or are available from the corresponding author upon request. The accession number for the raw sequencing data obtained from the MeRIP-seq reported in this paper is GEO: GSE125803 [https://www.ncbi.nlm.nih.gov/geo/query/acc.cgi]. The source data underlying Figures and Supplementary Figures are provided as a Source Data file.
