# Supplementary material for: Viral N6-methyladenosine upregulates replication and pathogenesis of human respiratory syncytial virus
Source: Nat Commun. 2019 Oct 9;10:4595. doi: 10.1038/s41467-019-12504-y (PMC6785563; doi:10.1038/s41467-019-12504-y)
Supplement: Supplementary file 1 — Supplementary Information [file 41467_2019_12504_MOESM1_ESM.pdf]

Viral N^6-methyladenosine upregulates replication and pathogenesis of human respiratory syncytial virus

Xue *et al*.

(A)

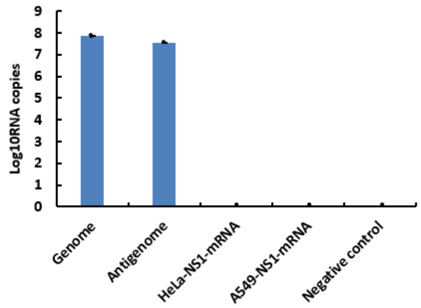

(B)

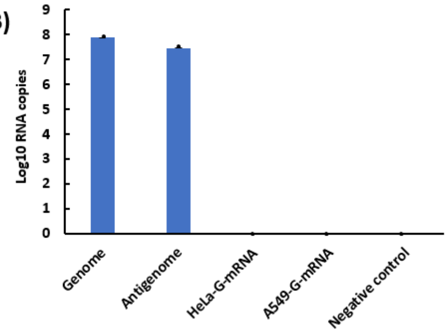

(C)

Sample	Average CT (β-actin)
Cell lysate	15.35 ±0.265
Purified virion from HeLa cells	Undetected
Purified virion from A549 cells	Undetected
Negative control	Undetected

(D)

Sample	Average CT (GAPDH)
Cell lysate	15.26 ±0.081
Purified virion from HeLa cells	Undetected
Purified virion from A549 cells	Undetected
Negative control	Undetected

Supplementary Fig. 1. Examination of the purity of virion RNA. Total RNA was extracted from highly purified RSV virions grown in HeLa cells as described in Materials and Methods. First strand DNA was synthesized by SuperScript III Reverse Transcriptase using Oligo d(T), followed by qPCR for quantification for viral mRNAs (NS1 and G) and host mRNA (β-actin and GAPDH). Results showed that virion RNA used for m^6A quantification and m^6A-seq were free of contamination of host RNAs and viral mRNAs. **(A) Quantification of NS1 mRNA**. Plasmid RW30 encoding the full-length cDNA of the RSV A2 strain was used to generate standard curve. Primers were designed to annealing to NS1 region in genome and antigenome. For positive control, RSV genome and antigenome from purified virion RNA were quantified by real-time RT-PCR targeting the NS1 gene. Distilled water was used as a negative control. NS1 gene was not detectable using cDNA synthesized from virion RNA using Oligo d(T). **(B) Quantification of G mRNA**. Primers were designed to amplify the G gene in real-time RT-PCR. Distilled water was used as a negative control. G gene was not detectable using cDNA synthesized from virion RNA using Oligo d(T). **(C) Quantification of β-actin mRNA**. The CT values were shown for each sample. Total host RNA from HeLa cells was served as a positive control. Distilled water was served as a negative control. β-actin mRNA was not detectable using cDNA synthesized from virion RNA using Oligo d(T). **(D) Quantification of GAPDH mRNA**. The CT values were shown for each sample. Total host RNA from HeLa cells was served as a positive control. Distilled water was served as a negative control. GAPDH mRNA was not detectable using cDNA synthesized from virion RNA using Oligo d(T). Data presented are the averages from three independent virion samples (n = 3).

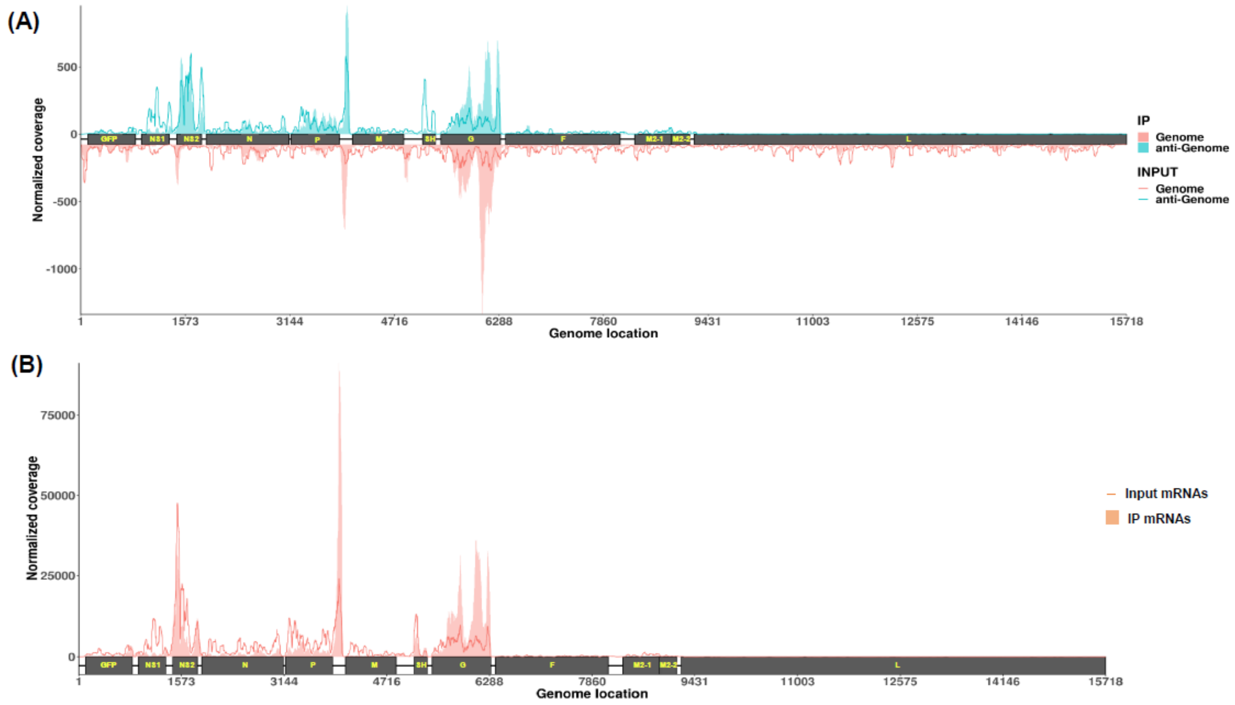

Supplementary Fig. 2. The RSV genome and antigenome/mRNAs are m^{6}A methylated. (A) Distribution of m^{6}A peaks in the RSV antigenome and genome of virions grown in HeLa cells. A schematic diagram of the RSV antigenome encoding 10 genes is shown. The normalized coverage from m^{6}A-seq of RSV RNA showing the distribution of m^{6}A-immunoprecipitated (IP) reads mapped to the RSV antigenome (blue block) and genome (pink block). The baseline distributions for antigenome and genome from input sample are shown as a blue and pink line respectively. Data presented are the averages from two independent virion samples (*n* = 2). **(B) Distribution of m^{6}A peaks in the RSV mRNAs from RSV-infected HeLa cells**. The distribution of m^{6}A-immunoprecipitated (IP) reads were mapped to the RSV mRNAs (pink block). The baseline distributions for mRNAs from input sample are shown as a pink line. Data presented are the averages from two independent virus-infected HeLa cell samples (*n* = 2). **(C) Distribution of m^{6}A peaks in the RSV antigenome and genome of virions grown in A549 cells**. Data presented are the averages from two independent virion samples (*n* = 2). **(D) Distribution of m^{6}A peaks in the RSV mRNAs from RSV-infected A549 cells**. Data presented are the averages from two independent virus-infected A549 cell samples (*n* = 2).

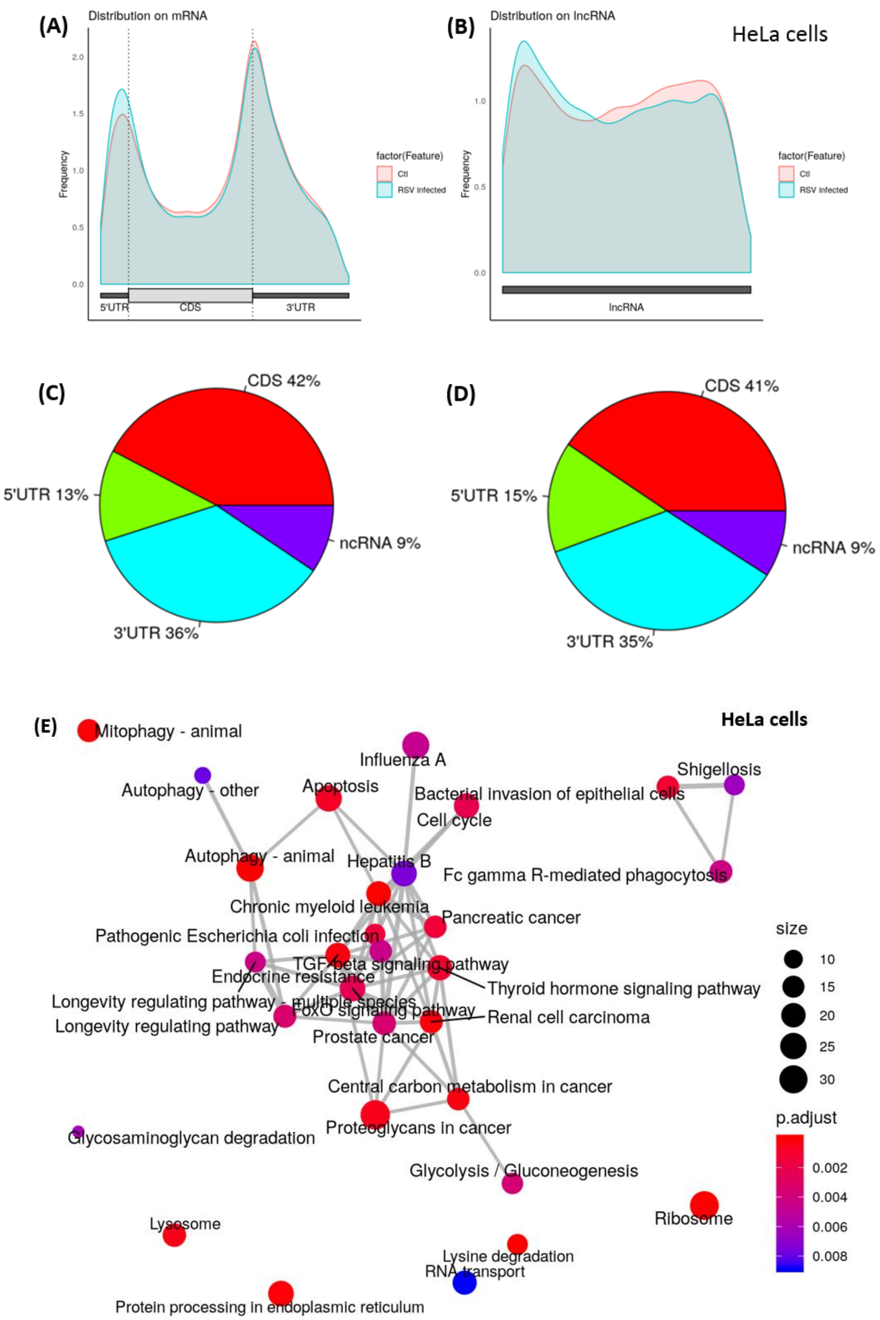

Supplementary Fig. 3. RSV infection alters the methylome of host transcripts in HeLa cells. Total RNAs were isolated from mock-infected and rgRSV-infected HeLa cells. Poly(A) enriched mRNAs were purified and subjected to m^{6}A-seq. **(A) Metagene analysis of m^{6}A peaks distribution along the human mRNA in control and infected HeLa cells. (B) Metagene analysis of m^{6}A peak distribution on lncRNA. (C and D) Distribution of m^{6}A peaks in the 5′ UTR, CDS, and 3′ UTR of host cell mRNA transcripts.** Charts show the proportion of m^{6}A peaks in the indicated regions in uninfected (**C**) and rgRSV-infected HeLa cells (**D**). **(E) GO graphs showing pathway clusters from differential expressed genes in rgRSV-infected HeLa cells.** Data presented are the averages from duplicate samples (*n* = 2).

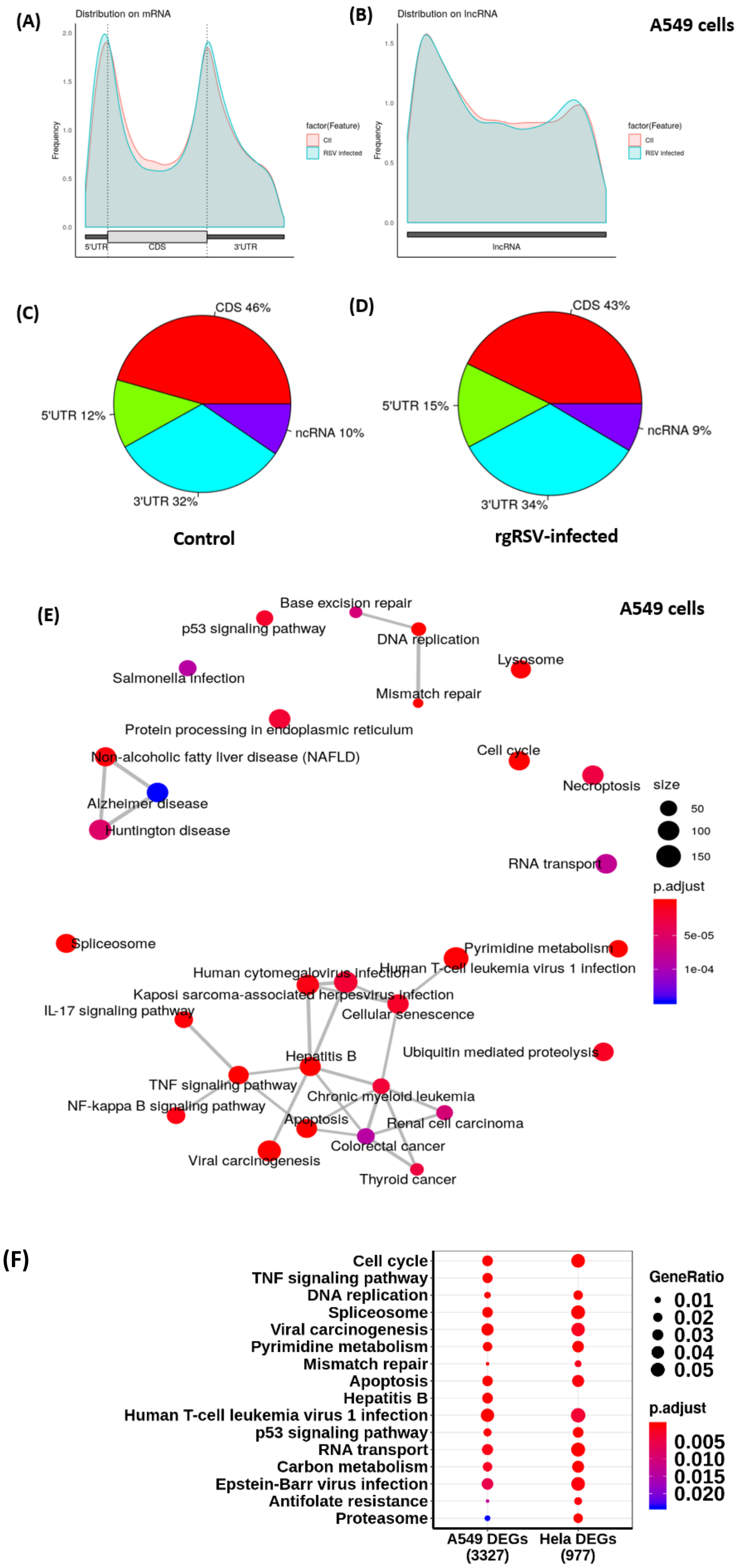
(A)
Distribution on mRNA
Frequency
5'UTR
CDS
3'UTR
factor(Feature)
Ctl
RSV infected
(B)
Distribution on lncRNA
lncRNA
A549 cells
(C)
CDS 46%
5'UTR 12%
ncRNA 10%
3'UTR 32%
Control
(D)
CDS 43%
5'UTR 15%
ncRNA 9%
3'UTR 34%
rgRSV-infected
(E)
A549 cells
Base excision repair
p53 signaling pathway
DNA replication
Lysosome
Salmonella infection
Mismatch repair
Protein processing in endoplasmic reticulum
Non-alcoholic fatty liver disease (NAFLD)
Cell cycle
Necroptosis
Alzheimer disease
Huntington disease
RNA transport
size
50
100
150
p.adjust
5e-05
1e-04
Spliceosome
Pyrimidine metabolism
Human cytomegalovirus infection
Human T-cell leukemia virus 1 infection
Kaposi sarcoma-associated herpesvirus infection
IL-17 signaling pathway
Cellular senescence
Ubiquitin mediated proteolysis
Hepatitis B
TNF signaling pathway
Chronic myeloid leukemia
NF-kappa B signaling pathway
Apoptosis
Renal cell carcinoma
Colorectal cancer
Viral carcinogenesis
Thyroid cancer
(F)
Cell cycle
TNF signaling pathway
DNA replication
Spliceosome
Viral carcinogenesis
Pyrimidine metabolism
Mismatch repair
Apoptosis
Hepatitis B
Human T-cell leukemia virus 1 infection
p53 signaling pathway
RNA transport
Carbon metabolism
Epstein-Barr virus infection
Antifolate resistance
Proteasome
A549 DEGs (3327)
Hela DEGs (977)
GeneRatio
0.01
0.02
0.03
0.04
0.05
p.adjust
0.005
0.010
0.015
0.020

Supplementary Fig. 4. RSV infection alters the methylome of host transcripts in A549 cells. Total RNAs were isolated from mock-infected and rgRSV-infected A549 cells. Poly(A) enriched mRNAs were purified and subjected to m^6A-seq. **(A) Metagene analysis of m^6A peak distribution along the human mRNA in control and infected A549 cells. (B) Metagene analysis of m^6A peak distribution in lncRNA. (C and D) Distribution of m^6A peaks in the 5′ UTR, CDS, and 3′ UTR of host cell RNA transcripts.** Charts show the proportion of m^6A peaks in the indicated regions in uninfected (**C**) and rgRSV-infected A549 cells (**D**). **(E) GO graphs showing pathway clusters from differential expressed genes in rgRSV-infected A549 cells.** Data presented are the average results from duplicate samples (*n* = 2). **(F) GO graphs showing pathway clusters from differential expressed genes which are overlapped between rgRSV-infected HeLa and A549 cells.**

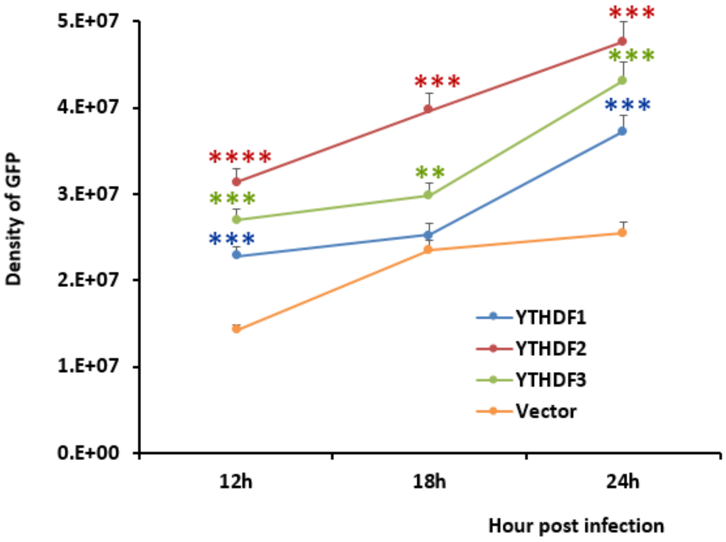

Supplementary Fig. 5. Overexpression of m^{6}A reader proteins enhances GFP density. The density of GFP expression in HeLa cells from **Fig.2B** was quantified by flow cytometry. Data presented are the averages from three independent experiments (n = 3). The P value (Student's t-test) for YTHDF1 at 12 and 24 h is ***P = 0.000409 and ***P = 0.000826 respectively; for YTHDF2 at 12, 18, and 24h is ****P =3.066×10^{-5}, ***P =0.000303, and ***P = 0.000122 respectively; for YTHDF3 at 12, 18, and 24h is ***P =0.000116, **P = 0.00606, and ***P =0.000274.

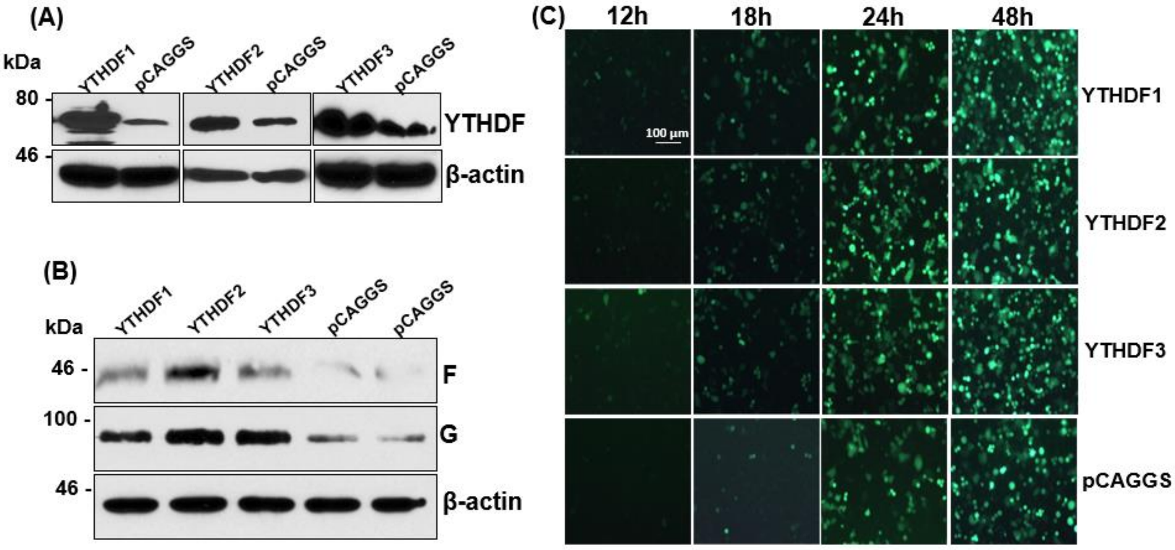

Supplementary Fig. 6. Transient expression of YTHDF1, 2, 3 proteins enhances RSV gene expression in HeLa cells. HeLa cells were transfected with 1 µg of plasmids encoding YTHDF1, 2, 3 or pCAGGS. At 36 h post-transfection, cells were infected with rgRSV at an MOI of 0.5. (A) Immunoblot analysis of YTHDF1, 2, 3 protein expression at 18 h post-infection. (B) Immunoblot analysis of RSV G and F protein expression at 18 h post-infection. (C) GFP expression. Data are from three independent experiments (*n* = 3). Micrographs with 10× magnification (scale bar of 100 µm) are shown. Western blots shown are representative of three independent experiments (*n* = 3).

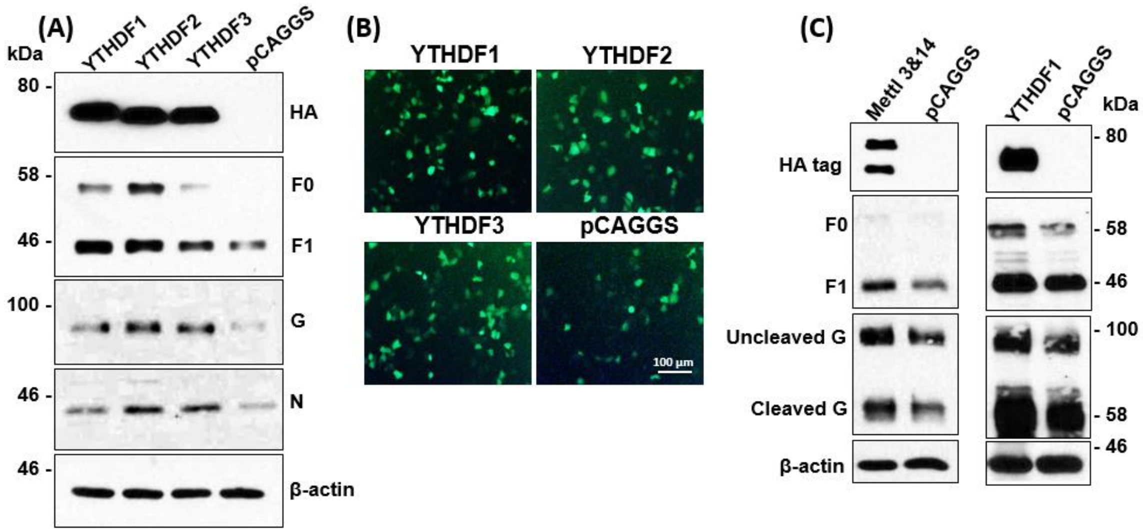

Supplementary Fig. 7. Transient expression of YTHDF1, 2, or 3 proteins enhances RSV gene expression in A549 and Vero cells. A549 or Vero cells were transfected with 1 µg of plasmid. At 36 h post-transfection, cells were infected with rgRSV at an MOI of 0.5. (A) Immunoblot analysis of RSV F, G, N, and HA-tagged reader proteins in A549 cells. (B) Dynamics of GFP expression in YTHDF1, 2 or 3 transfected A549 cells at 24 h post-infection. Micrographs with 10× magnification (scale bar of 100 µm) are shown. (C) Immunoblot analysis of RSV G and F proteins in Vero cells. The m^6A reader protein (YTHDF1) and writer proteins (METTL3 and METTL14) were used for transfection. Data are the representatives of three independent experiments (n = 3).

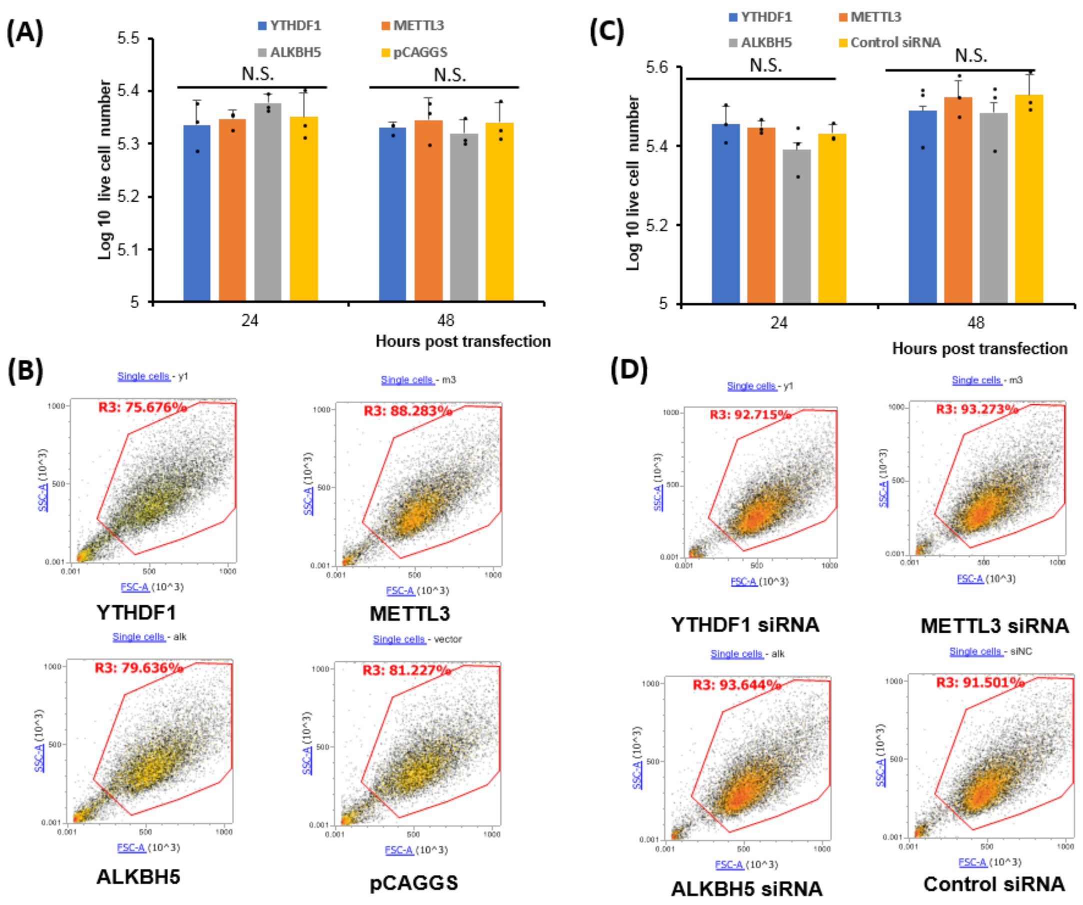

Supplementary Fig. 8. The effects of m^{6}A reader and writer proteins on cell growth or metabolism. (A) The effect of overexpression of m^{6}A-related protein on cell growth. A549 cells were transfected with 1 µg of plasmids encoding a representative gene of m^{6}A reader (YTHDF1), writer (METTL3), and eraser (ALKBH5) proteins. At 24 and 48 h post-transfection, cells were trypsinized and counted by flow cytometry. Flow cytometry data are plotted as mean of 3 independent experiments ± standard deviation. **(B) Raw flow cytometry plot at 48 h. (C) The effect of knockdown of m^{6}A-related protein on cell growth.** A549 cells were transfected with control siRNA or siRNA targeting a representative gene of m^{6}A reader (YTHDF1), writer (METTL3), and eraser (ALKBH5) proteins. Cell count from flow cytometry at 24 and 48 h. **(D) Raw flow cytometry cell counts at 48 h post transfection.** N.S. indicates no significant differences among groups.

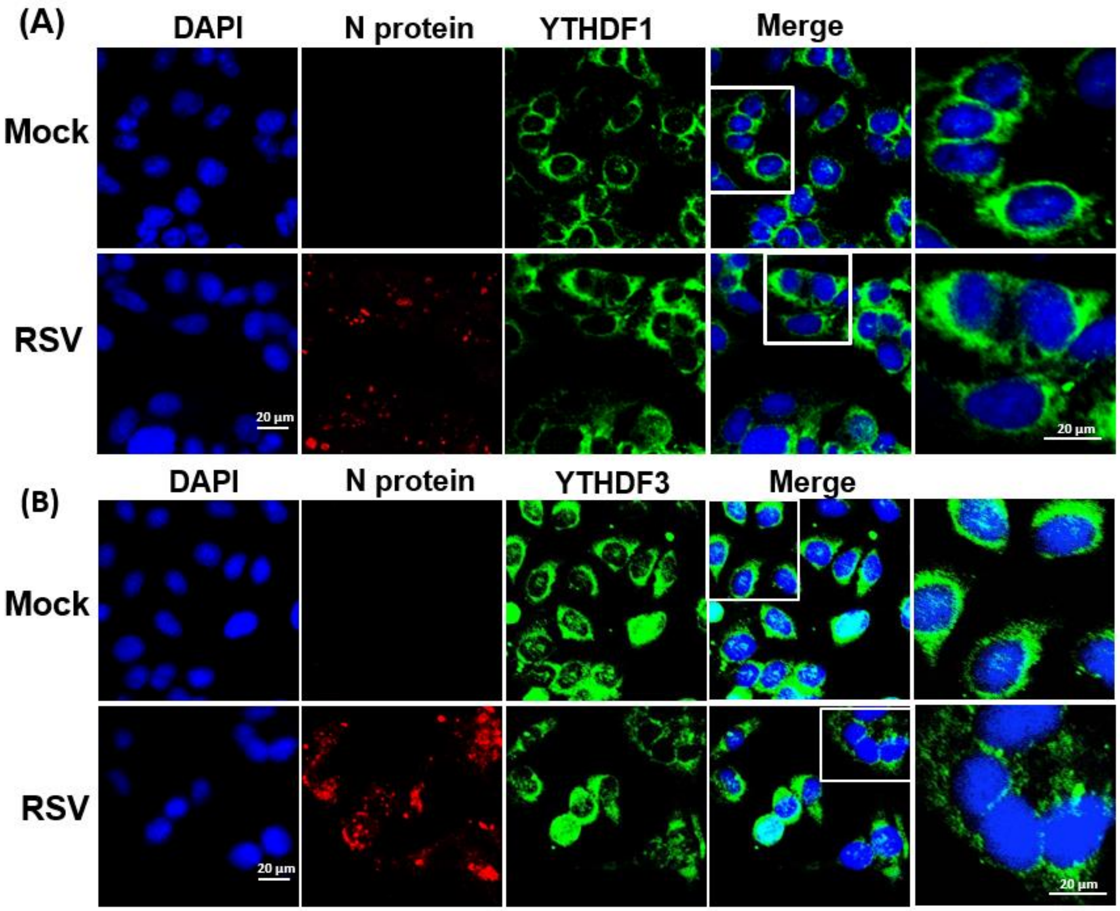

Supplementary Fig. 9. Distribution of m^{6}A reader proteins YTHDF1 and 3 in mock and RSV-infected HeLa cells. HeLa cells were infected with rgRSV at an MOI of 10. At 24 h post-infection, mock- or rgRSV-infected cells were stained with anti-reader antibody (green) and anti-RSV N protein antibody (red), and analyzed by confocal microscopy. Nuclei (blue) were labeled with DAPI. Micrographs with 60× magnification (scale bar of 20 µm) are shown. **(A) Reader protein YTHDF1; and (B) Reader protein YTHDF3.** Representative images are shown from three independent experiments (*n* = 3).

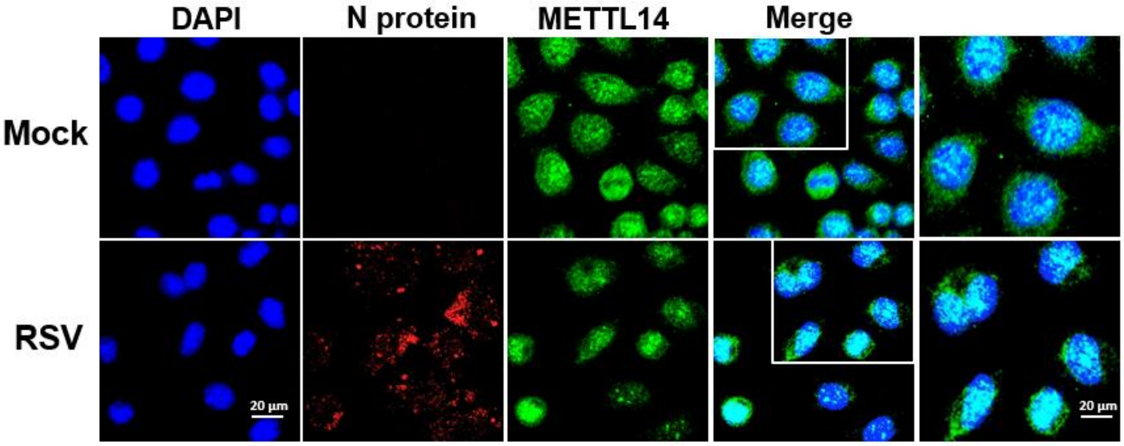

Supplementary Fig. 10. Distribution of m^{6}A writer protein METTL14 in mock and RSV-infected HeLa cells. HeLa cells were infected with rgRSV at an MOI of 10. At 24 h post-infection, mock- or rgRSV-infected cells were stained with anti-writer antibody (green) and anti-RSV N protein antibody (red) and analyzed by confocal microscopy. Micrographs with 60× magnification (scale bar of 20 μm) are shown. Nuclei (blue) were labeled with DAPI. Representative images are shown from three independent experiments (n = 3).

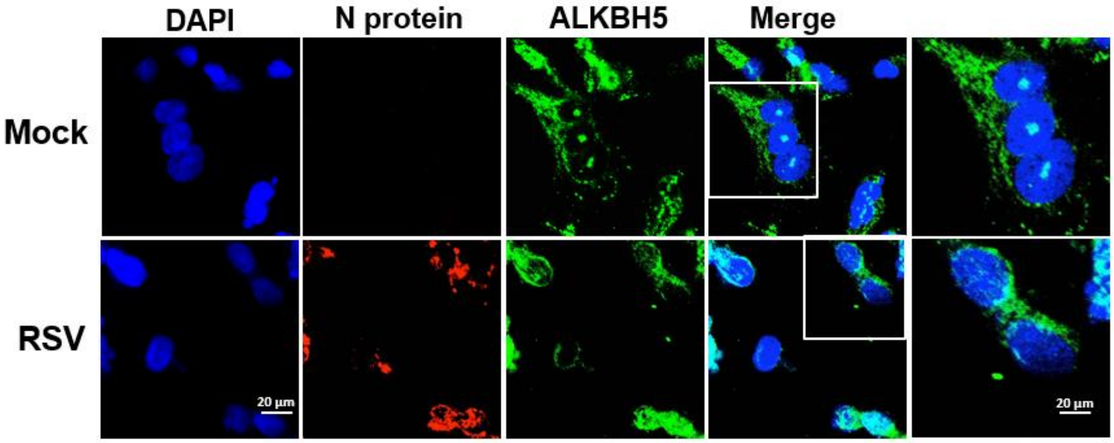

Supplementary Fig. 11. Distribution of m^{6}A eraser protein ALKBH5 in mock and RSV-infected HeLa cells. HeLa cells were infected with rgRSV at an MOI of 10. At 24 h post-infection, mock- or rgRSV-infected cells were stained with anti-eraser antibody (green) and anti-RSV N protein antibody (red) and analyzed by confocal microscopy. Micrographs with 60× magnification (scale bar of 20 μm) are shown. Nuclei (blue) were labeled with DAPI. Representative images are shown from three independent experiments (*n* = 3).

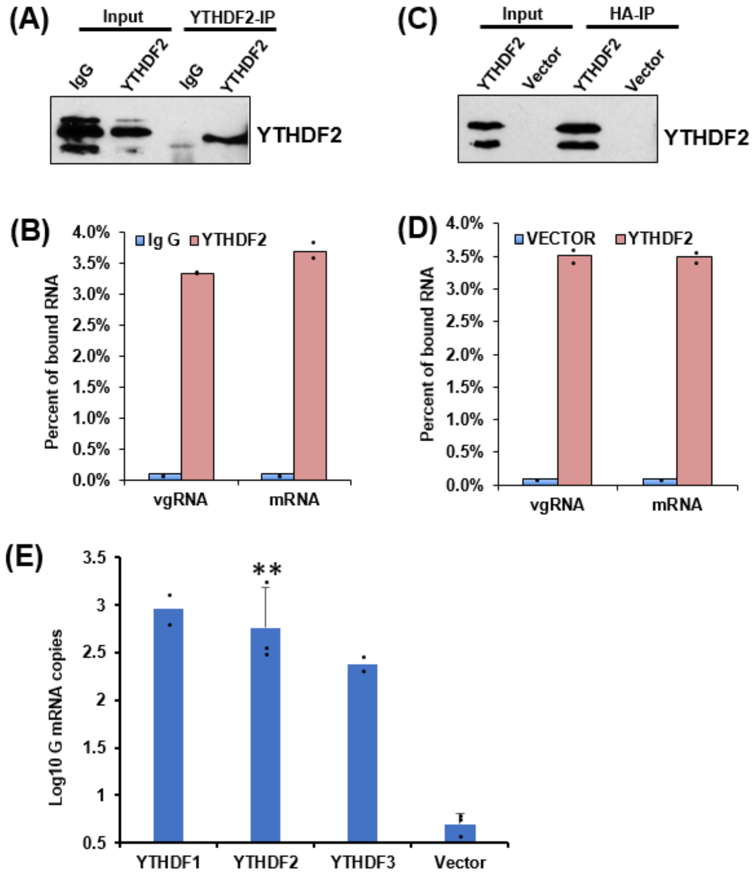

Supplementary Fig. 12. m^{6}A reader protein binds to RSV genomic RNA and mRNA. HeLa cells stably expressing YTHDF2 and vector control HeLa cells were infected with rgRSV at an MOI of 1.0. At 24 h post-infection, cells were lysed and cytoplasmic extracts were immunoprecipitated with an antibody against YTHDF2 (A) or an equivalent amount of HA-tag (non-specific IgG control) (C). Data are representative of two independent experiments (n = 2). The amount of vgRNA and mRNA captured by the YTHDF2 antibody (B) or the HA-tag antibody (D) was quantified by real-time RT-PCR, as was the input RNA, and graphed as the percentage of input. Data are the average of two independent experiments (n = 2). (E) m^{6}A reader proteins bind to G mRNA. HeLa cells stably expressing YTHDF1, 2, and 3 and vector control HeLa cells were infected with rgRSV at an MOI of 1.0. At 24 h post-infection, cells were lysed and cytoplasmic extracts were immunoprecipitated with an antibody against HA-tag antibody. The amount of G mRNA bound to YTHDF1, 2, and 3 was quantified by real-time RT-PCR. Data for YTHDF1 and YTHDF3 are the average of two independent experiments (n = 2). Data for YTHDF2 and vector are the average of three independent experiments (n = 3). The P value (Student's t-test) for YTHDF2 is $^{**}P = 0.00122$.

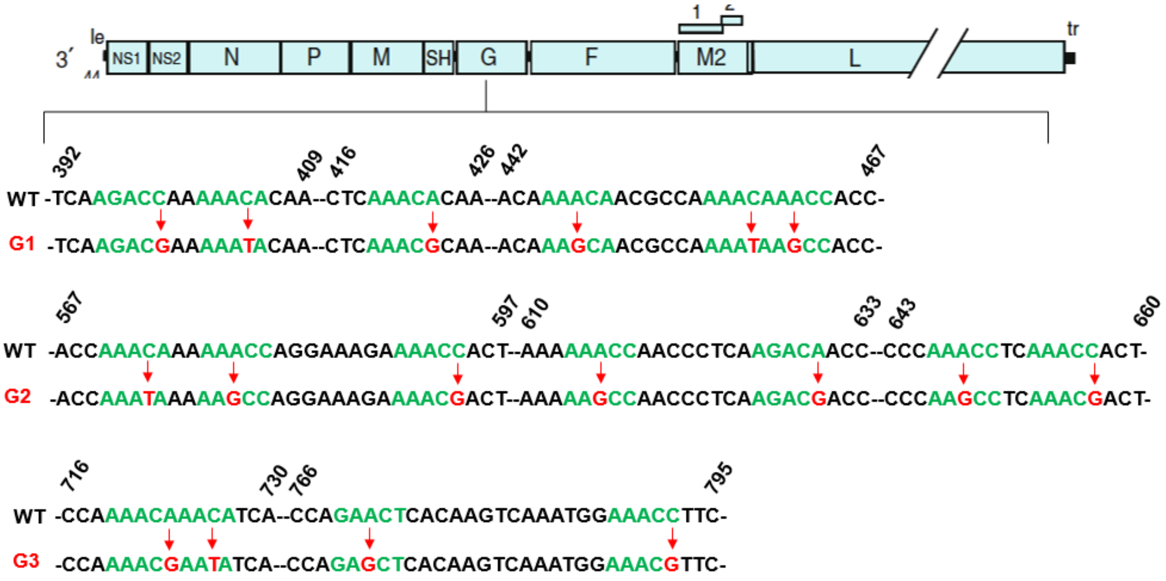

Supplementary Fig. 13. Mutagenesis strategy in putative m^{6}A site in the RSV G mRNA. Schematic diagram of the RSV genome with the mutations for altering the critical A or C residues in the m^{6}A motifs (red arrows) to produce rgRSV lacking that putative m^{6}A modification site in the G gene. Three m^{6}A peaks, G1, G2, and G3, are shown; each containing 6, 7, and 4 m^{6}A sites, respectively. Consensus m^{6}A motifs (green) and inactivating mutations (red) are shown. Dashes represent nucleotides not shown. G gene sequence of RSV A2 strain (accession number M74568) is shown.

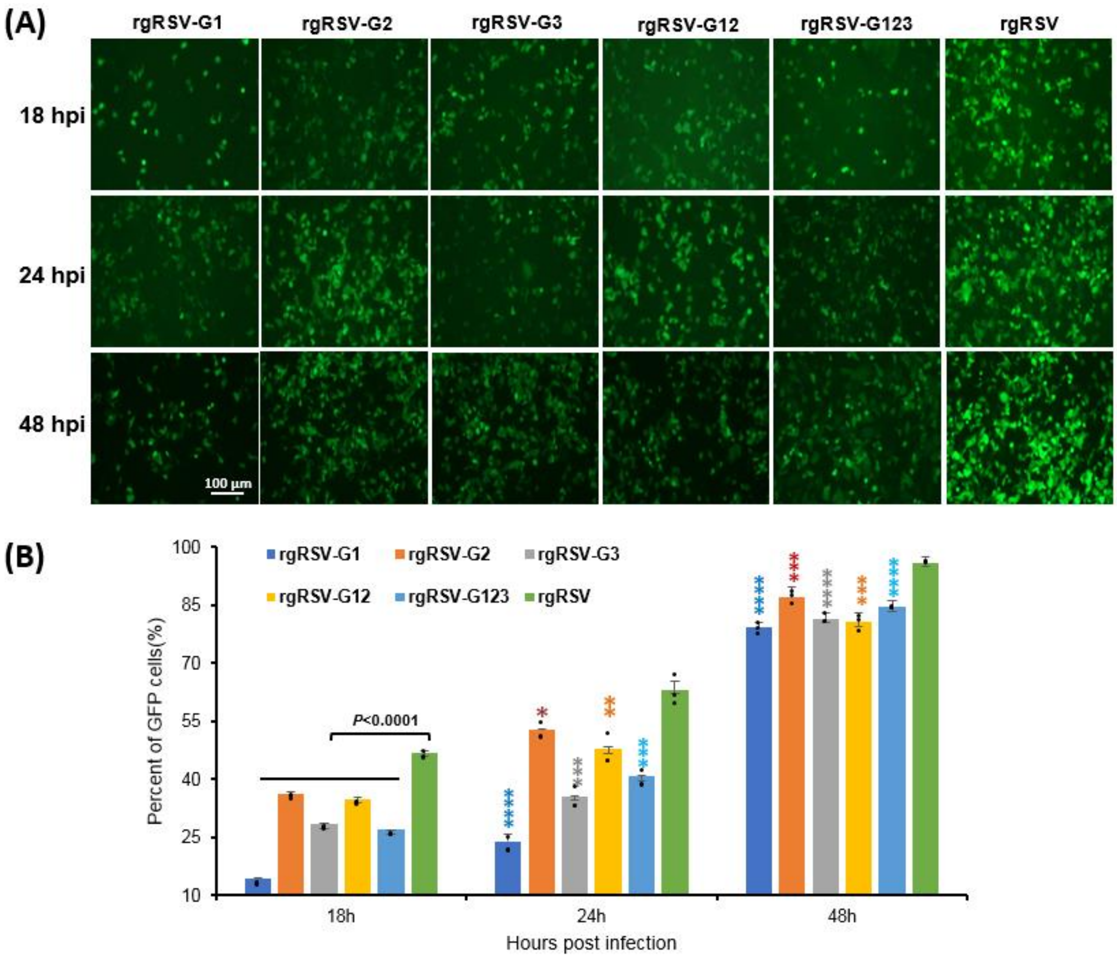

Supplementary Fig. 14. Replication of m^{6}A mutated rgRSVs in A549 cells. (A) GFP expression of m^{6}A mutated rgRSVs. Confluent A549 cells were infected with each m^{6}A-deficient rgRSV mutant at an MOI of 1.0, GFP images were photographed at 18, 24, and 48 h post-infection**.** Micrographs with 10× magnification (scale bar of 100 µm) are shown. **(B) Quantification of GFP-positive cells by flow cytometry.** GFP images shown are representative of three independent experiments (n = 3). Flow cytometry data are expressed as mean of three independent experiments ± standard deviation. The P value (Student's t-test) for rgRSV-G1 at 18, 24, and 48 h is ***$P = 6.12\times10^{-7}$, ****$P = 8.67\times10^{-5}$, and ****$P = 4.12\times10^{-5}$, respectively; for rgRSV-G2 at 18, 24, and 48 h is ****$P = 6.89\times10^{-5}$, *P = 0.0144 and ***P =0.000644, respectively; for rgRSV-G3 at 18, 24, and 48 h is ****$P = 6.61\times10^{-6}$, ***P = 0.000545, and ****$P = 2.09\times10^{-5}$; for rgRSV-G12 at 18, 24, and 48 h is ****$P = 3.58\times10^{-5}$, **P =0.00796, and ***P =0.000167, respectively; for rgRSV-G123 at 18, 24, and 48 h is ****$P = 4.35\times10^{-6}$, ***P = 0.000848, and ****$P = 6.35\times10^{-7}$, respectively.

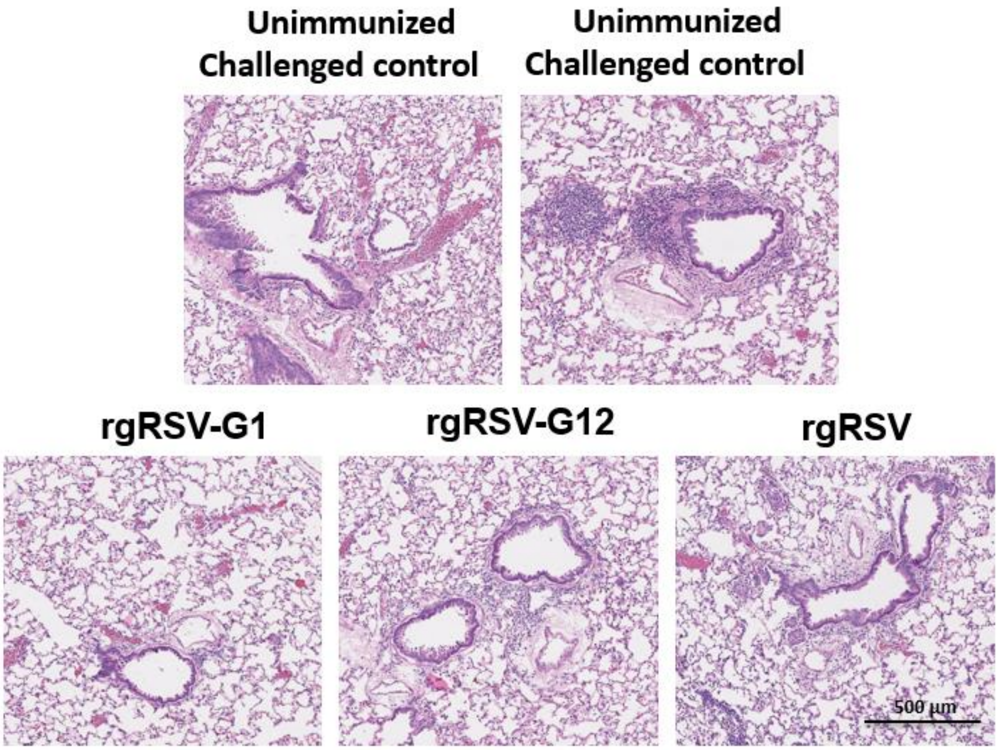

Supplementary Fig. 15. Immunization with m^{6}A mutated rgRSVs protects cotton rats from lung damage after RSV challenge. Four-week-old SPF cotton rats were inoculated intranasally with 2.0×10^5 $TCID_{50}$ of each rgRSV. At week 4 post-immunization, cotton rats (*n* = 5) were challenged with 2.0×10^5 $TCID_{50}$ rgRSV. At day 4 post-challenge, the cotton rats were sacrificed, and right lung lobe of each cotton rat was fixed in 4% neutral buffered formaldehyde, embedded in paraffin, sectioned at 5 µm, and stained with hematoxylin-eosin (HE) for the examination of histological changes by light microscopy. Representative pathological changes from each group are shown. Micrographs with 20× magnification (scale bar of 500 μm) are shown.

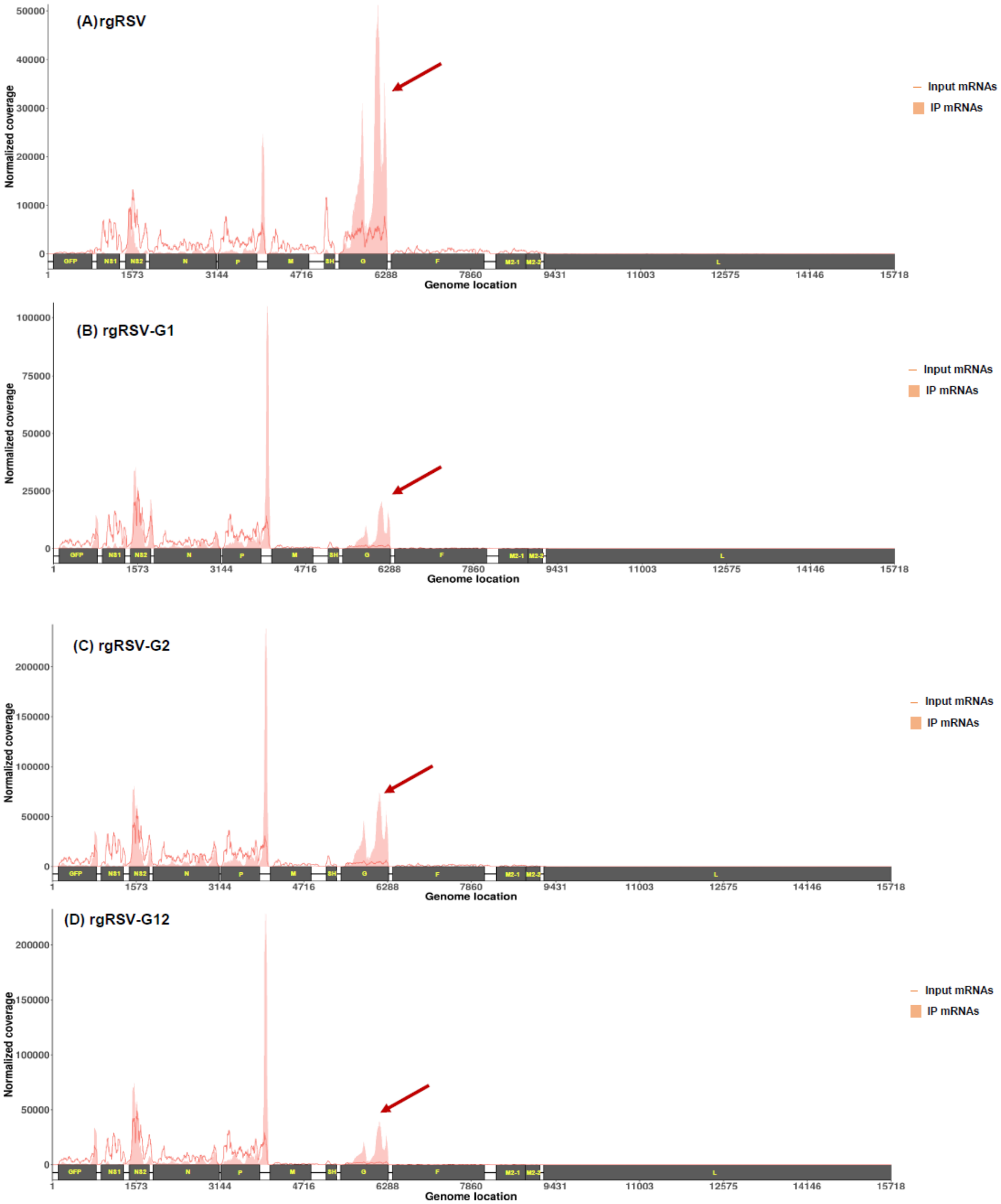

Supplementary Fig. 16. Distribution of m^{6}A peaks on the RSV mRNAs from A549 cells infected by m^{6}A-mutated rgRSVs. Confluent A549 cells were infected by each m^{6}A-deficient rgRSV at an MOI of 1.0, cell lysates were harvested at 36 h post-infection. Total RNAs were extracted from cell lysates, and were enriched for mRNA by binding to oligo dT, and subjected to m^{6}A-seq. The distribution of m^{6}A-immunoprecipitated (IP) reads were mapped to the RSV mRNAs (pink block). The baseline distributions for mRNAs from input sample are shown as a pink line. Data presented are the mean coverage from two independent virus-infected A549 cell samples (n = 2). Red arrow indicates the m^{6}A enrichment in G mRNA.

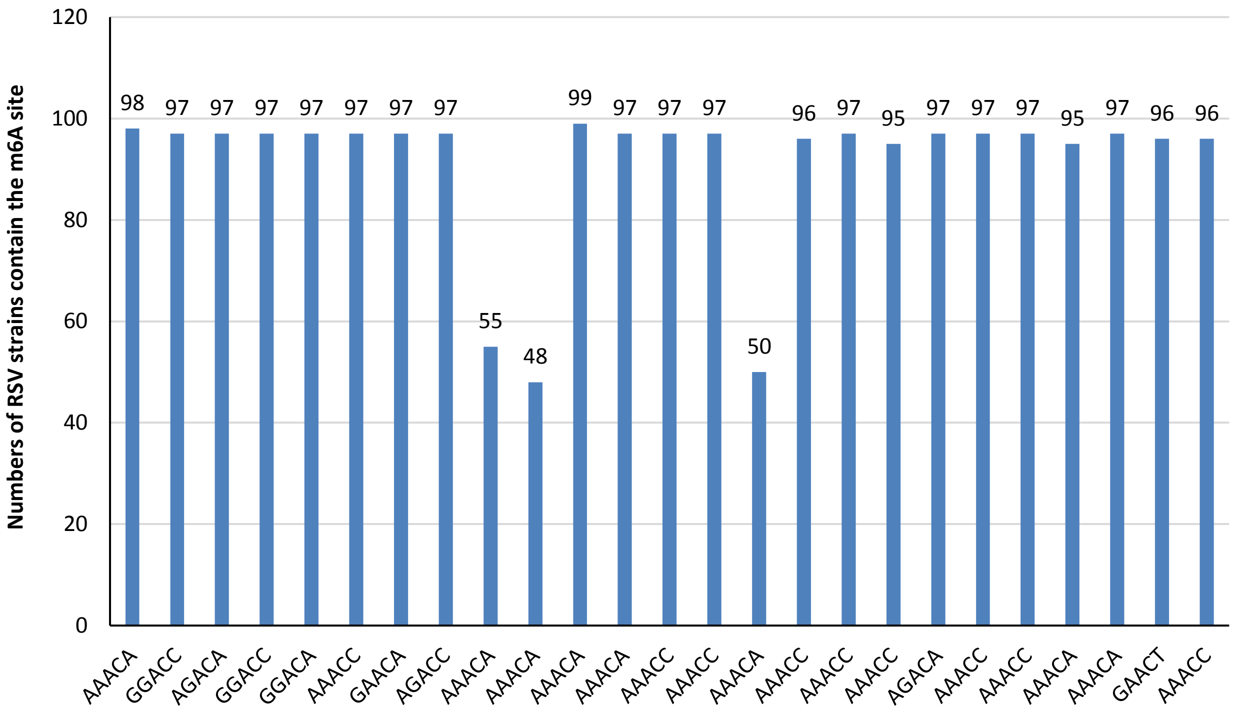

Supplementary Fig.17. Conserved m^{6}A sites in different RSV strains. Based on the m^{6}A-seq data from rgRSV-infected A549 cells, the G mRNA has a total of 25 putative m^{6}A sites. 100 RSV strains with full-length G mRNA available in GenBank were selected for sequence alignment. Conserved m^{6}A sites in these 100 RSV strains were identified. X axis indicates the 25 putative m^{6}A sites. Y axis indicates the numbers of RSV strains containing this specific m^{6}A site in X axis.

Supplementary Tables

Supplementary Table 1, m^{6}A peaks in RSV RNAs purified from virions grown in HeLa cells

RSV RNAs[A]	Peak no.	Peak range (nt)[B]	Gene location[C]	Peak size (nt)	Enrichment Score	Enrichment Fold[D]
Genome	1	599-972	*gs*, NS2	373	5.07	2.34
	2	1571-1645	N	74	3.42	1.77
	3	1795-1944	N	149	2.89	1.53
	4	2617-2766	P	149	3.65	1.87
	5	3066-3215	*ig*	149	8.81	3.13
	6	3963-4262	M, *ge*, *ig*	299	3.22	1.68
	7	4711-5533	G, *ge*, *ig*	822	6.68	2.74
	8	11291-1365	L	74	3.39	1.76
	9	13459-3533	L	74	3.29	1.71
	10	13758-3832	L	74	2.70	1.43
	11	13908-3982	L	74	3.44	1.78
Antigenome	1	1645-1719	N	74	2.39	1.26
	2	2543-2991	P	448	3.07	1.62
	3	4786-5458	G	672	5.49	2.45
	4	5833-5907	F	74	4.66	2.22

A. The rgRSV harboring GFP between leader and NS1 gene is used for m^{6}A-seq. One m^{6}A peak is detected in GFP region in genome: peak range = 721-780, peak size = 60nt, enrichment score = 2.06; enrichment fold = 1.44. No m^{6}A peak is found in GFP region in antigenome.
B. Nucleotide sequence is referred to RSV A2 strain. Nucleotide ranges are indicated. m^{6}A peaks in G gene region are highlighted by yellow color.
C. The RSV genes and regulatory elements are covered by m^{6}A peaks. These regions may contain m^{6}A sites. However, whether these regions indeed contain m^{6}A sites will require to search the presence of m^{6}A motif, Pu [G>A]m^{6}AC[A/C/U] motif (Pu represents purine). RSV gene start, gene end, and intergenic sequence are indicated by *gs*, *ge*, and *ig*, respectively.
D. log2 enrichment of the m^{6}A peaks identified in RSV antigenome and genome.

Supplementary Table 2, m^{6}A peaks in RSV mRNAs from rgRSV-infected HeLa cells

RSV RNAs	Peak no.	Peak range (nt) [A]	Gene location [B]	Peak size (nt)	Enrichment Score	Enrichment Fold [C]
mRNAs	1	1-50	Leader	49	3.87	1.95
	2	450-648	NS1, *ge*, *ig*, *gs*, NS2	198	3.45	1.78
	3	898-997	NS2, *ge*, *ig*	99	5.45	2.44
	4	1895-1944	N	49	2.79	1.48
	5	2194-2243	N	49	5.73	2.52
	6	2444-2542	P	98	3.24	1.69
	7	2743-2792	P	49	4.04	2.01
	8	3042-3190	P, *ge*, *ig*	148	16.05	4.01
	9	3740-3789	M	49	3.70	1.88
	10	3989-4138	M, *ge*, *ig*	149	5.95	2.57
	11	4288-4337	*gs*, SH	49	3.81	1.93
	12	4388-4437	SH	49	7.81	2.96
	13	4687-5533	G, *ge*, *ig*	846	15.38	3.94
	14	7279-7328	*ge*, *ig*	49	4.48	2.16
	15	8326-8375	*ig*, *gs*	49	8.32	3.05
	16	13909-3958	L	49	5.43	2.44

A. Nucleotide sequence is referred to RSV A2 strain. Nucleotide ranges are indicated. m^{6}A peaks in G gene region are highlighted by yellow color. No m^{6}A peak is found in GFP mRNA.
B. The RSV mRNAs and regulatory elements are covered by m^{6}A peaks. These regions may contain m^{6}A sites. However, whether these regions indeed contain m^{6}A sites will require to search the presence of m^{6}A motif, Pu [G>A]m^{6}AC[A/C/U] motif (Pu represents purine). RSV gene start, gene end, and intergenic sequence are indicated by *gs*, *ge*, and *ig*, respectively.
C. log2 enrichment of the m^{6}A peaks identified in RSV mRNAs.

Supplementary Table 3, m^6A peaks in RSV RNAs purified virions grown in A549 cells

RSV RNAs[A]	Peak no.	Peak range (nt)	Gene location	Peak size (nt)	Enrichment Score	Enrichment Fold
Genome	1	599-798	*gs*, NS2	199	13.73	3.77
	2	1746-1944	N	198	21.94	4.45
	3	2194-2243	N	49	10.15	3.34
	4	2444-2542	P	98	7.62	2.93
	5	2593-2841	P	248	9.65	3.27
	6	2992-3240	P, *ge*, *ig*, *gs*	248	178.31	7.47
	7	4737-5085	G	348	35.42	5.14
	8	5135-5483	G	348	90.27	6.49
	9	5883-6082	F	199	6.30	2.65
Antigenome	1	400-848	NS1, *ge*, *ig*, *gs*, NS2	448	9.89	3.31
	2	898-997	NS2, *ge*, *ig*	99	6.66	2.73
	3	1347-1446	N	99	4.57	2.19
	4	1496-1695	N	199	6.86	2.77
	5	2095-2243	N	148	14.49	3.85
	6	2344-2393	P	49	3.07	1.62
	7	2444-3290	P, *ge*, *ig*, *gs*, M	846	20.56	4.36
	8	4039-4138	*Ig*	99	8.43	3.07
	9	4537-4686	*ig*, *gs*, G	149	3.64	1.86
	10	4737-5434	G	697	47.63	5.57
	11	5484-5533	*Ig*	49	17.78	4.15
	12	5783-6082	F	299	12.5	3.64
	13	8326-8375	*ig*, *gs*	49	5.55	2.47
	14	12114-2263	L	149	5.78	2.53
	15	14806-4855	L	49	13.29	3.73

A: The rgRSV harboring GFP between leader and NS1 gene is used for m^6A-seq. One m^6A peak is detected in GFP region in antigenome: peak range = 705-794, peak size = 89nt, enrichment score = 16.4; enrichment fold = 4.05. No m^6A peak is found in GFP region in genome.

Supplementary Table 4, m^{6}A peaks in RSV mRNAs purified from rgRSV-infected A549 cells

RSV RNAs[A]	Peak no.	Peak range (nt)	Gene location	Peak size (nt)	Enrichment Score	Enrichment Fold
**mRNAs**	1	1-50	leader	49	3.86	1.95
	2	450-798	NS1, *ge*, *ig*, *gs*, NS2	348	12.19	3.61
	3	898-997	NS2, *ge*, *ig*	99	3.58	1.84
	4	1048-1246	N	198	2.58	1.37
	5	1297-1396	N	99	3.03	1.60
	6	1496-1695	N	199	5.12	2.35
	7	1796-1994	N	198	5.87	2.55
	8	2045-2642	N, *ge*, *ig*, *gs*, P	597	5.21	2.38
	9	2743-3589	P, *ge*, *ig*, *gs*, M	846	22.23	4.47
	10	3989-4138	M, *ge*, *ig*	149	4.34	2.11
	11	4188-4337	*ig*, *gs*, SH	149	2.46	1.30
	12	4487-5533	*ig*, *gs*,G, *ge*, *ig*	1046	85.69	6.42
	13	5783-6082	F	299	6.61	2.72
	14	6481-6580	F	99	2.42	1.27
	15	7628-7876	M2-1	248	2.86	1.51
	16	7977-8026	M2-1	49	2.90	1.53
	17	8326-8424	*ig*, *gs*, L	98	2.54	1.34
	18	12114-12163	L	49	5.10	2.35

A: The rgRSV harboring GFP between leader and NS1 gene is used for m^{6}A-seq. One m^{6}A peak is detected in GFP mRNA: peak range = 735-794, peak size = 39nt, enrichment score = 1.97; enrichment fold = 1.40.

Supplementary Table 5, Overlapping m^{6}A peaks in RSV RNAs purified virions grown in HeLa and A549 cells

RSV RNAs	Overlapping Peak no.	Peak range (nt)	Peak size (nt)	Gene location
Genome	1	599-798	199	*gs*, NS2
	2	1795-1944	149	N
	3	2617-2766	149	P
	4	3066-3215	149	*ig*
	5	4737-5085	348	G
	6	5135-5483	348	G
Antigenome	1	1645-1695	50	N
	2	2543-2991	448	P
	3	4786-5434	648	G
	4	5833-5907	74	F

Supplementary Table 6, Overlapping m⁶A peaks in RSV mRNAs purified from rgRSV-infected HeLa and A549 cells

RSV RNAs	Overlapping Peak no.	Peak range (nt)	Peak size (nt)	Gene location
mRNAs	1	1-50	49	Leader
	2	450-648	198	NS1, *ge*, *ig*, *gs*, NS2
	3	898-997	99	NS2, *ge*, *ig*
	4	1895-1944	49	N
	5	2194-2243	49	N
	6	2444-2542	98	P
	7	2743-2792	49	P
	8	3989-4138	149	M, *ge*, *ig*
	9	4288-4337	49	*gs*, SH
	10	4687-5533	846	G, *ge*, *ig*
	11	8326-8375	49	*ig*, *gs*

Supplementary Table 7 siRNA used for knocking down host m^{6}A machinery

siRNA	Sequences (5’-3’)
YTHDF1	5’-CCGCGTCTAGTTGTTCATGAA-3’
YTHDF2	5’-AAGGACGTTCCCAATAGCCAA-3’
YTHDF3	5’-ATGGATTAAATCAGTATCTAA-3’
METTL3	5’-CTGCAAGTATGTTCACTATGA-3’
METTL14	5’-AAGGATGAGTTAATAGCTAAA-3’
ALKBH5	5’-AAACAAGTACTTCTTCGGCGA-3’
FTO	5’-AAATAGCCGCTGCTTGTGAGA-3’
Control siRNA	5’ ACGTGACACGTTCGGAGAA-3’

Supplementary Table 8 Primers used in this study

Primer names	Purpose	Sequences (5'-3')
Genome-F	Quantify viral Genome/Antigenome	5'-AAATGCGTACAACAAACTTGCATAA-3'
Genome-R	Quantify viral Genome/Antigenome	5'-AATATTTCCTTTTCCACAACCTTCC-3'
NS1-F	Quantify NS1 mRNA	5'-CAATTCATTGAGTATGATAAAAGTTAGATTACA-3'
NS1-R	Quantify NS1 mRNA	5'-AATATTATTATTAGGGCAAATATCACTACTTGTA-3'
G-F	Quantify G mRNA	5'-AGGACCAACGCACCGCTAAG-3'
G-R	Quantify G mRNA	5'-GTTCTTGATCTGGCTTGTTGCATC-3'
Oligo-dT	RT mRNA	5'-TTTTTTTTTTTTTTTTTT-3'
F5810-5816	Mutagenesis for m^6A	5'-CCACAACAGTCAAGACGAAAAATACAACAACAACTCAAAC-3'
R5810-5816	Mutagenesis for m^6A	5'-GTTTGAGTTGTTGTTGTATTTTTCGTCTTGACTGTTGTGG-3'
F5834	Mutagenesis for m^6A	5'-ACAACAACAACTCAAACGCAACCCAGCAAGC-3'
R5834	Mutagenesis for m^6A	5'-CTTGCTGGGTTGCGTTTGAGTTGTTGTTGTG-3'
F5858-5870-5873	Mutagenesis for m^6A	5'-CCCACCACAAAGCAACGCCAAAATAAGCCACCAAGCAAAC-3'
R5858-5870-5873	Mutagenesis for m^6A	5'- GTTTGCTTGGTGGCTTATTTTGGCGTTGCTTTGTGGTGGG-3'
F5984-5990-6005	Mutagenesis for m^6A	5'-CAAAAGAATACCAAATAAAAAGCCAGGAAAGAAAACGACTACCAAGCCCAC-3'
R5984-5990-6005	Mutagenesis for m^6A	5'- GTGGGCTTGGTAGTCGTTTTCTTTCCTGGCTTTTTATTTGGTATTCTTTTG-3'
F6026-6041	Mutagenesis for m^6A	5'-CCACAAAAAAGCCAACCCTCAAGACGACCAAAAAAGATC-3'
R6026-6041	Mutagenesis for m^6A	5'-GATCTTTTTTGGTCGTCTTGAGGGTTGGCTTTTTTGTGG-3'
F6059-6068	Mutagenesis for m^6A	5'-CAAAAAAGATCCCAAGCCTCAAACGACTAAATCAAAGG-3'
R6059-6068	Mutagenesis for m^6A	5'-CCTTTGATTTAGTCGTTTGAGGCTTGGGATCTTTTTTG-3'
F6134-6137	Mutagenesis for m^6A	5'-CATCAACACCACCAAAACGAATATCATAACTACACTACTC-3'
R6134-6137	Mutagenesis for m^6A	5'- GAGTAGTGTAGTTATGATATTCGTTTTGGTGGTGTTGATG-3'
F6182-6203	Mutagenesis for m^6A	5'-CAGGAAATCCAGAGCTCACAAGTCAAATGGAAACGTTCCACTCAACTTC-3'
R6182-6203	Mutagenesis for m^6A	5'- GAAGTTGAGTGGAACGTTTCCATTTGACTTGTGAGCTCTGGATTTCCTG-3'
