# Supplementary material for: Viral N6-methyladenosine upregulates replication and pathogenesis of human respiratory syncytial virus
Source: Nat Commun. 2019 Oct 9;10:4595. doi: 10.1038/s41467-019-12504-y (PMC6785563; doi:10.1038/s41467-019-12504-y)
Supplement: Supplementary file 2 — Reporting Summary [file 41467_2019_12504_MOESM2_ESM.pdf]

nature research

Corresponding author(s): Jianrong Li

Last updated by author(s): Aug 15, 2019

Reporting Summary

Nature Research wishes to improve the reproducibility of the work that we publish. This form provides structure for consistency and transparency in reporting. For further information on Nature Research policies, see Authors & Referees and the Editorial Policy Checklist.

Statistics

For all statistical analyses, confirm that the following items are present in the figure legend, table legend, main text, or Methods section.

n/a	Confirmed	
☐	☒	The exact sample size (*n*) for each experimental group/condition, given as a discrete number and unit of measurement
☐	☒	A statement on whether measurements were taken from distinct samples or whether the same sample was measured repeatedly
☐	☒	The statistical test(s) used AND whether they are one- or two-sided<br>*Only common tests should be described solely by name; describe more complex techniques in the Methods section.*
☐	☒	A description of all covariates tested
☐	☒	A description of any assumptions or corrections, such as tests of normality and adjustment for multiple comparisons
☐	☒	A full description of the statistical parameters including central tendency (e.g. means) or other basic estimates (e.g. regression coefficient) AND variation (e.g. standard deviation) or associated estimates of uncertainty (e.g. confidence intervals)
☐	☒	For null hypothesis testing, the test statistic (e.g. *F*, *t*, *r*) with confidence intervals, effect sizes, degrees of freedom and *P* value noted<br>*Give P values as exact values whenever suitable.*
☒	☐	For Bayesian analysis, information on the choice of priors and Markov chain Monte Carlo settings
☐	☒	For hierarchical and complex designs, identification of the appropriate level for tests and full reporting of outcomes
☒	☐	Estimates of effect sizes (e.g. Cohen's *d*, Pearson's *r*), indicating how they were calculated

Our web collection on statistics for biologists contains articles on many of the points above.

Software and code

Policy information about availability of computer code

Data collection	All Western blot images were scanned and collected using Adobe photoshop version 7. All ELISA-based data were recored by ELISA plate reader. The flow cytometry data were collected using Attune NxT Flow Cytometer software. All immunofluorescence images were collected using an Olympus FV 1000 confocal microscopy system at The Ohio State University Campus Microscopy & Imaging Facility or Image J at Nationwide Children's Hospital. All original data were kept in a notebooks or electronically in lab computers.
Data analysis	Nucleotide and amino acid sequence analysis was performed using DNAstar Software (Lasergene, Madison, Wisconsin). For m6A sequencing data analysis, after removing the adapter sequences, the m6A reads were mapped to the human genome (hg38) and rgRSV genome and antigenome by using Hisat2 (Kim et al., 2015) with peak calling as described (Ma et al., 2017). Metagene analysis was performed by R package Guitar (Cui et al., 2016). Differential methylation analysis was performed with count based negative binomial model implemented in QNB test (Liu et al., 2017). Gene Ontology (GO) analysis was performed using the GO Enrichment Analysis tool from the Gene Ontology Consortium. GO graphs were plotted using the Web server REVIGO (Supek et al., 2011). All biological experiments were at done three replicates (three independent experiments) to warrant the samples size and statistical analysis, as standard for biological experiments. Results were reported as average ± standard deviation, as indicated in the figures. Data (such as Western blot gels, immunofluorescence images) are representative of three independent experiments. Statistical analysis was performed by one-way multiple comparisons using SPSS (version 8.0) statistical analysis software (SPSS Inc., Chicago, IL). A P value of <0.05 was considered statistically significant.*P<0.05, **P<0.01, ***P<0.001, ****P<0.0001.

For manuscripts utilizing custom algorithms or software that are central to the research but not yet described in published literature, software must be made available to editors/reviewers. We strongly encourage code deposition in a community repository (e.g. GitHub). See the Nature Research guidelines for submitting code & software for further information.

Data

Policy information about availability of data

All manuscripts must include a data availability statement. This statement should provide the following information, where applicable:

- Accession codes, unique identifiers, or web links for publicly available datasets
- A list of figures that have associated raw data
- A description of any restrictions on data availability

All materials, reagents, and data reported in this manuscript are available from the corresponding author upon request.

Field-specific reporting

Please select the one below that is the best fit for your research. If you are not sure, read the appropriate sections before making your selection.

☒ Life sciences ☐ Behavioural & social sciences ☐ Ecological, evolutionary & environmental sciences

For a reference copy of the document with all sections, see nature.com/documents/nr-reporting-summary-flat.pdf

Life sciences study design

All studies must disclose on these points even when the disclosure is negative.

Sample size	Figs.1 and 2 are m6A sequencing results. Duplicated samples (including RNAs from virions, virus-infected cells, mock-infected cells) were submitted to m6A-sequencing. All other experiments were at done three replicates (three independent experiments) to warrant the samples size and statistical analysis, as standard for biological experiments. Results were reported as average ± standard deviation, as indicated in the figures. Western blot gels and immunofluorescence images are representative of three independent experiments. Quantitative analysis of Western blot was performed by either densitometric scanning of autoradiographs or by using a phosphorimager (Typhoon; GE Healthcare, Piscataway, NJ) and ImageQuant TL software (GE Healthcare, Piscataway, NJ). Statistical analysis was performed by one-way multiple comparisons using SPSS (version 8.0) statistical analysis software (SPSS Inc., Chicago, IL). A P value of <0.05 was considered statistically significant.*P<0.05, **P<0.01, ***P<0.001, ****P<0.0001.
Data exclusions	No data was excluded in the analysis.
Replication	All attempts at the replication were successful. The results were reliably reproduced.
Randomization	All samples/organisms were randomly assigned to experimental groups. Data were also collected randomly. Bulk stocks of plasmids, antibody, virus stocks, and other reagents were prepared for each experiments. The cells were randomly assigned to each treatment group. Animal experiments were randomized (see below for detail).
Blinding	Investigators were blinded to group allocation for animal experiments, including virus inoculation, tissue collection, processing, virology analysis, and evaluation of histologic lesions. Investigators were also blinded to for in vitro experiments during data collection and analysis.

Reporting for specific materials, systems and methods

We require information from authors about some types of materials, experimental systems and methods used in many studies. Here, indicate whether each material, system or method listed is relevant to your study. If you are not sure if a list item applies to your research, read the appropriate section before selecting a response.

Materials & experimental systems

n/a	Involved in the study	
☐	☒	Antibodies
☐	☒	Eukaryotic cell lines
☒	☐	Palaeontology
☐	☒	Animals and other organisms
☒	☐	Human research participants
☒	☐	Clinical data

Methods

n/a	Involved in the study	
☒	☐	ChIP-seq
☐	☒	Flow cytometry
☒	☐	MRI-based neuroimaging

Antibodies

Antibodies used	The below antibodies were validated for using in several assays such Western blot, Immunofluorescence assay.<br>YTHDF1: Proteintech, catlog #17479-1-AP<br>YTHDF2: EMD Minipore, catlog # ABE 542<br>YTHDF3: Abcam, catlog# ab103328<br>Mettl3: Proteintech, catlog # 15073-1-AP

Mettl14: Abcam, catlog # ab98166
ALKBH5: Sigma, catlog # HPA007196
FTO: Abcam, catlog # ab124892
Anti-RSV- Serum: Virostat, catlog # 0601
Anti-RSV-F/G: available from co-author Dr. Mark Peeples' lab.
Anti-Flag: Sigma, catlog # F1804-50UG
Beta-actin: Abcam, catlog # ab8227
Beta-tublin: Abcam, catlog # ab6046
Anti-HA: Abcam, catlog # ab49969

Validation

All antibodies (except anti-RSV F and G antibody) were purchased from commercial companies and have been validated by the companies. The anti-RSV F and G antibodies were available from co-author Dr. Mark Peeples' lab and validated by several assays including Western blot, ELISA, and immunostaining.

Eukaryotic cell lines

Policy information about cell lines

Cell line source(s)

HeLa (ATCC CCL-2), A549 (ATCC CCL-185), Vero (ATCC CRL-CCL81), and HEp-2 (ATCC CCL-23) were purchased from American Type Culture Collection (ATCC, Manassas, VA). Primary, well-differentiated human airway epithelial cells (HAE) were prepared by co-author Dr. Mark Peeples' lab in the Nationwide Children Hospital. HAE culture is primary cell culture from lungs of health human donors under IRB protocol no. IRB-009-02.

Authentication

HeLa, Vero, A549, and HEp-2 cells were purchased from ATCC. According to ATCC's instruction, the cell lines have been authenticated before shipped to us. We first made a bulk stocks for each cell line after recovering from the original frozen vial from ATCC. We will discard the cells after passing 15-20 passages, and recover new cells from frozen stocks. Cell morphology was monitored at each passage by microscope. Periodically, growth curve analysis for cell line was performed.

Mycoplasma contamination

Periodically, all cell lines in our lab were tested for contamination of mycoplasma by the LookOut Mycoplasma PCR Detection Kit (Sigma, MP0035-1KT). All cell lines used in this study were free of mycoplasma.

Commonly misidentified lines
(See ICLAC register)

No commonly used misidentified cell lines were used.

Animals and other organisms

Policy information about studies involving animals; ARRIVE guidelines recommended for reporting animal research

Laboratory animals

Cotton rats (Sigmodon hispidus) have been shown to be a highly permissive small animal model for RSV. The animal protocol (No. 2009A0221) was approved by the Institutional Laboratory Animal Care and Use Committee (ILACUC) at The Ohio State University (OSU). 6-week-old female SPF cotton rats were purchased from Envigo, Indianapolis, Indianan, USA, and housed in BSL-2 animal care facility at OSU, which is AAALAC accredited. For RSV infection experiments, groups of five animals were randomly formed. Each group was inoculated with RSV mutant. All analysis (such as tissue collection, processing, virus detection, histologic evaluation) were blinded to researchers. Viral titers in nasal turbinate and lung were average of five animals ± standard deviation, and were statistically analyzed.

Wild animals

N/A

Field-collected samples

N/A

Ethics oversight

The animal protocol (No. 2009A0221) and all procedures were approved and supervised by the Institutional Laboratory Animal Care and Use Committee (ILACUC) at The Ohio State University (OSU).

Note that full information on the approval of the study protocol must also be provided in the manuscript.

Flow Cytometry

Plots

Confirm that:

- [ ] The axis labels state the marker and fluorochrome used (e.g. CD4-FITC).
- [ ] The axis scales are clearly visible. Include numbers along axes only for bottom left plot of group (a 'group' is an analysis of identical markers).
- [ ] All plots are contour plots with outliers or pseudocolor plots.
- [x] A numerical value for number of cells or percentage (with statistics) is provided.

Methodology

Sample preparation

HeLa cells were infected by rgRSV (RSV expressing GFP) at an MOI ranging from 0.1 to 1.0. Mock-infected cells were used as controls. At 12, 24, and 48 h post-infection, cells were trypsinized and fixed by 4% paraformaldehyde solution, and GFP positive cells were analyzed by flow cytometry.

Instrument	Attune NxT Flow Cytometer (ThomoFisher Scientific).
Software	Attune NxT software was used to collect and analyze the data.
Cell population abundance	Single population of A549 or HeLa cells (purchased from ATCC) were used for rgRSV infection. Percent of GFP positive or negative cells were calculated.
Gating strategy	We only have a single parameter (which is the GFP marker) for our RSV infection experiments. The cells were either GFP positive or negative after rgRSV (RSV expressing GFP) infection. First of all, we analyzed the mock-infected cells, which are GFP negative. These cells were gating controls (GFP negative cells). Then, we ran the rgRSV-infected cells, which showed number of GFP-positive and negative cells using the gating controls. The percent of GFP positive cells were calculated. Results are the averages from three independent experiments and are expressed as mean ± standard errors.

☒ Tick this box to confirm that a figure exemplifying the gating strategy is provided in the Supplementary Information.
