# Supplementary material for: Viral N6-methyladenosine upregulates replication and pathogenesis of human respiratory syncytial virus
Source: Nat Commun. 2019 Oct 9;10:4595. doi: 10.1038/s41467-019-12504-y (PMC6785563; doi:10.1038/s41467-019-12504-y)
Supplement: Supplementary file 3 — Description of Additional Supplementary Files [file 41467_2019_12504_MOESM3_ESM.pdf]

**Description of Additional Supplementary Files**

File Name: Supplementary Data 1
Description: Differentially $m^6$ A methylated peaks in HeLa cells after RSV infection. A total of 2256 differentially $m^6$ A methylated peaks are identified using count based QNB test

File Name: Supplementary Data 2
Description: Differentially expressed genes in HeLa cells after RSV infection. Over nine thousands differentially expressed genes are identified by analysis of RNA-seq data of host cell (HeLa) at adjusted P value cutoff of 0.05.

File Name: Supplementary Data 3
Description: Differentially expressed genes in A549 cells after RSV infection. Over seven thousands differentially expressed genes are identified by analysis of RNA-seq data of host cell (A549) at adjusted P value cutoff of 0.05

File Name: Supplementary Data 4
Description: Differentially $m^6$ A methylated peaks in A549 cells after RSV infection. Differentially $m^6$ A methylated peaks are identified using count based QNB test.
